# A comparative study of four physical education curricula on the developmental behavior of Chinese preschool children aged 4 to 6 years and its correlation with balance ability

**DOI:** 10.3389/fpubh.2025.1477001

**Published:** 2025-03-10

**Authors:** Minjie Qiao, Laite Yu, Jingyu Shi, Xiaoting Wang, Ruiyuan Li, Zicheng Wan, Dongsheng Lu

**Affiliations:** ^1^Shaanxi Provincial Children and Adolescent Physical Education Research Center, Xi’an, China; ^2^School of Physical Education, Shaanxi Normal University, Xi’an, China; ^3^Department of Physical Education, Henan Vocational College of Water Conservancy and Environment, Zhengzhou, China

**Keywords:** developmental behavior, gross motor, balance ability, correlation, preschool children, physical education curriculum

## Abstract

Early childhood development is important for the future developmental behavior, physical fitness, and social adaptation. The content of the physical education curriculum in kindergarten is crucial for the growth of preschool children. The aim of this study was to explore the effects of 12-week of 4 different physical education curriculum interventions on developmental behavior and balance ability, and the correlation between these two factors in preschool children. 94 preschool children aged 4–6 years were stratified and randomly assigned to tennis group (TG), football group (FG), sensory integration group (SIG), and control group (CG). All interventions resulted in greater improvements in all developmental behavior indicators and balance ability after intervention compared to baseline. The SIG showed greater improvements in total development quotient (DQ), gross motor DQ, fine motor DQ, and balance ability than the other three groups after intervention. No significant difference in balance ability between FG and SIG was found. There was a positive and significant correlation between adaptability DQ, social behavior DQ, and balance ability after SIG intervention. The SIG training could correlate children’s balance ability with their social behavior and adaptability. The sensory integration curriculum might be the optimal curriculum to promote the efficient improvement of preschool children’s developmental behavior and motor competence.

## 1 Introduction

Children and adolescents have demonstrated a detrimental impact on the future development of motor competence and rapid progress due to the lack of appropriate physical training during the critical stage of growth and development (1, 2). This is especially true for preschool children aged 4–6 years old (2, 3). Early childhood is an important period for rapid development of body and brain. Therefore, timely training of gross motor skills and fine motor skills is crucial for enhancing physical fitness, motor skills, and physical health (4, 5). According to the latest figures released by Ministry of Education of People’s Republic of China, 46.2755 million young children have received preschool education in kindergarten in 2023, 1.7766 million fewer than in 2022 (6). The sharp decrease in the number of young children attending kindergarten has highlighted the crucial role of maintaining high-quality early childhood education curricula. Tennis, soccer and sensory integration movement include both gross and fine motor skills, and are dominated by the upper limbs, lower limbs, and the whole body coordination, respectively. Previous researches suggested that tennis, soccer and sensory integration courses were more beneficial than traditional physical education courses (4, 7). However, no study has concurrently compared the effects of the aforementioned three courses.

The physical education curriculum at kindergarten has been suggested as one of the most important and available avenues for the promotion of physical activity, physical fitness, and motor skills among preschool children (8). Chinese young children spend approximately 40 h each week in kindergarten, where they are provided with ample opportunities for physical activities and structured physical education classes (9). Studies have found that young children’s physical activity in the early years was associated with a range of positive health outcomes (e.g., cognitive development, psychosocial, fundamental motor skill and balance ability), and there was a significant positive correlation between early physical activity and balance ability, object control skills, and gross motor skills (10, 11). Therefore, it is necessary for preschool children to carry out effective physical education curriculum and activity at kindergarten. In previous studies, the effects of various factors such as gender, age, health status, intervention, duration of intervention on young children development have been explored (2, 12–16). However, few studies have examined the impacts of different physical education curriculum on young children development.

The Developmental Scale for Children aged 0–6 years (DSC) is a standard diagnostic assessment tool widely used in China to assess the developmental behavior level of children aged 0–6 years (17, 18). The DSC represents the latest version of the Children Neuropsychological and Behavior Scale (CNBS), encompassing five attributes (gross motor, fine motor, language, adaptability, and social behavior) while also providing measures for mental age (MA) and development quotient (DQ). Different versions of DSC have been extensively employed within academic research (17, 19, 20). For example, in the research of Li et al. (20). the CNBS-R2016 and Griffiths Mental Development Scales for China were both employed to evaluate the development of children with autism spectrum disorder, and the results showed good consistency in the developmental assessment. Furthermore, the result verified the reliability of CNBS-R2016. Nevertheless, the application of DSC has not been found yet.

Balance is fundamental to perform gross motor skills. It is an essential prerequisite to promote physical health and motor skills in preschool children (21). Balance ability is defined as the ability to maintain a certain body posture under dynamic or static conditions, including static balance and dynamic balance (22). Currently, an increasing number of studies have investigated the correlation between gross motor skills and balance abilities in preschool children (12, 13, 23). For example, in the research of Jiang et al. (12) it was found that both the dynamic and static balance abilities of young children aged 3–6 years exhibited a positive correlation with their gross motor skills. Furthermore, the correlation between object control skills, physical activity, physical fitness, gender, and age with balance ability have also been evaluated (10, 13, 24, 25). However, the current research on the correlation between the indicators in the DSC and balance ability has not been found. Additionally, previous researches have indicated that preschool children can enhance their dynamic and static balance by engaging in physical education curriculum such as taekwondo, Chinese martial arts, gymnastics, and tennis (26–28). These studies confirmed the positive effect of different physical education curriculum on prompting balance ability. However, no studies have concurrently investigated the influence of tennis, football, and sensory integration curriculum on balance ability or the correlation between the developmental behavior of these three curricula and their balance ability.

Based on the information above, this study aimed to explore the impacts of a 12-week different physical education curriculum on the developmental behavior of preschool children aged 4 to 6 in China and its correlation with their balance ability. Four physical education curricula with structured and game-based were adopted: tennis, football, sensory integration and traditional physical education. It was hypothesized that the three intervention curricula would result in greater improvements in total development quotient (DQ), gross motor DQ, fine motor DQ, language DQ, adaptability DQ, social behavior DQ, and balance ability compared to traditional curriculum. Specifically, the sensory integration curriculum intervention may be the optimal physical education curriculum.

## 2 Materials and methods

### 2.1 Participants recruitment

Based on the experimental design, the sample size was estimated prospectively using G*power v3.1.0 (Franz Faul, University of Kiel, Germany) with a level of 0.05 and power of 0.80. The effect size was set at 0.35 based on a previous study (29). As a result, the sample size was estimated to be 96 subjects. To allow for study withdrawal and dropout, we decided to recruit an additional 4 participants. Thus, the planned sample size of this study was 100.

Participates were 4–6 years old preschool children (male = 50, female = 50) recruited from a kindergarten in Xi’an city, China. To ensure the universality and trustworthiness of this study, the allocation adopted a stratified random method. Participants were initially stratified by sex (male or female) and subsequently randomized into four groups with a 1:1:1:1 allocation ratio, namely: Tennis Group, Football Group, Sensory Integration Group, and Control Group (each group comprising N total = 25, with N male or female =12/13). However, six participates were discarded due to physical discomfort and temporary leave (male = 4, female = 2). A total of 94 participates were finally enrolled in this study. The inclusion criteria included (1) be healthy with no developmental delay or chronic diseases; (2) no cognitive impairment and understand and follow instructions; (3) be able to participate in all intervention processes; (4) did not participate in any other physical training except kindergarten physical education curriculum. Table 1 presents the demographic characteristics of the final sample. Informed written consent was obtained from all participants’ parents before the experiment. The study received ethical approval from the Ethics Committee of Shaanxi Normal University (approval number: 202416028).

**Table 1:** Baseline demographic characteristics of the participants.

	TG	FG	SIG	CG
N children	24 (25)	23 (25)	22 (25)	25 (25)
N male	12 (13)	12 (13)	10 (13)	12 (13)
N female	12 (12)	11 (12)	12 (13)	13 (13)
Ages (years)	4.75 ± 0.51	4.80 ± 0.49	4.59 ± 0.43	4.60 ± 0.5
Height (cm)	111.71 ± 6.52	112.30 ± 7.99	110.95 ± 7.76	112.80 ± 8.44
Body weight (kg)	20.08 ± 3.39	20.65 ± 3.75	19.59 ± 4.20	20.04 ± 3.43

TG, Tennis Group; FG, Football Group; SIG, Sensory Integration Group; CG, Control Group; N (N), actual number (allocated number). Data were represented as Mean ± Standard Deviation (SD).

### 2.2 Experimental design

**Figure 1:**
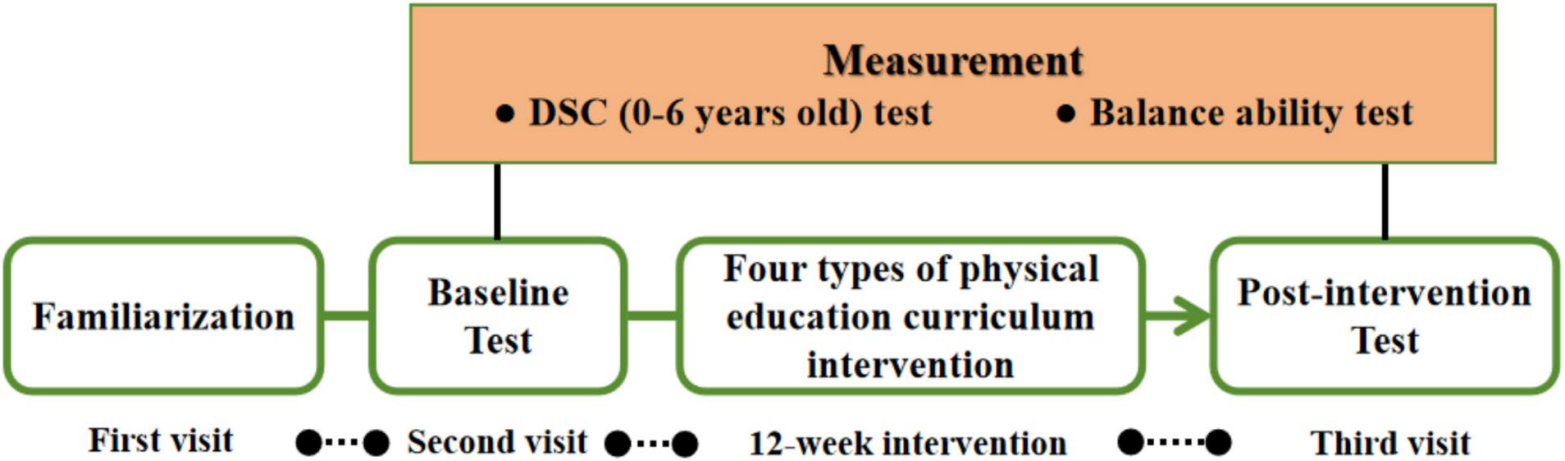
Experimental design.

This study was designed as a four-group parallel randomized controlled trial lasted between 6 March and 30 May in 2023. All the testing and intervention were performed in a kindergarten stadium. Before formal experiment, each participant was instructed to familiar with the test and intervention protocol. According to the aforementioned groups, participants in the three experimental groups received structured curriculum interventions in tennis, soccer, and sensory integration, while participants in the control group received traditional physical education curriculum. All interventions consisted of three training sessions per week for 12 weeks with each session lasting 30 min. The physical education curriculum interventions will be carried out by researchers with teaching experience. Moreover, two additional researchers (three from each group) will be assigned to each group to ensure the safety and quality of the intervention. The developmental behavior test and balance ability test were assessed at baseline and post-intervention. The experimental design is schematized in Figure 1.

### 2.3 Intervention programme

Each curriculum of the three experimental groups and the control group was based on structured lessons, which consist of a 5-min warm-up, 20 min main content exercises, and a 5-min cool-down activity. The warm-up consisted of light-intensity movements (e.g., wrist rotation, and leg swing), moderate-intensity activities (e.g., arm rotation, lunges with rotation and knee-up walk), and higher-intensity activities (e.g., arm sprint, dynamic stretching, on-site running, and high knees); cool-down started with moderate-intensity activities and ended with light-intensity movements (9). The main part of the lesson included 5 min of moderate-to-vigorous intensity activity according to the respective intervention curriculum, with at least 2 min of vigorous-intensity activity, every 10 min (9, 30). The contents of the main part in tennis and football group were replaced every 4 weeks to achieve a progressive learning and training. The main part of the control group followed a kindergarten-based physical education curriculum, including rhythm exercises, group games, gymnastics, and free play (4). The three interventions and control group were carried out in the form of games to increase children’s interest, and only differed in the main part (see Supplementary Appendix 1). The movements and contents of all groups were simple and there was no discernible division of difficulty levels. The protocol and components of the curriculum interventions were refined by verbal consulting with experts and teachers in preschool education. These interventions were carried out within the physical education curriculum plan of kindergartens, to avoid extra curriculum for the preschool children in the intervention groups.

### 2.4 Measurements

#### 2.4.1 Balance ability measures

The specific contents and scoring criteria of the balance test was performed with reference to published literature (31). The validity of this balance test has been verified among young children aged 3–6 years in China (31, 32). The content of the balance test was set according to the *Guidelines for the Learning and Development of Children aged 3–6* (33) and the *Guidelines for Kindergarten Education* (Trial) issued (34) by the ministry of Education of the People’s Republic of China, which made this test more scientific and credible. The balance test contained five items: stand on 1 foot, walk 3 meters on the balls of feet, walk 5 meters on the balls of feet, stand on 1 foot with eyes closed, and balance beam. The scoring criteria of the balance test differed between male and female participants and were categorized into 3 different age bands (3–4, 4–5, and 5–6 years). The specific tests, directions, materials and site layout, cautions and scoring criteria of the balance test were based on the published article (31). In this study, the final balance score was obtained by averaging the results of the scores from two balance test items in the same age bands. The specific contents and scoring criteria of the balance test were shown in Table 2.

**Table 2:** The specific contents and scoring criteria of the balance test.

Rating scale/score		3–4 Years		4–5 Years		5–6 Years	
		Stand on one foot/s	Walk 3 meters on the balls of feet/s	Walk 5 meters on the balls of feet/s	Stand on one foot with eyes closed/s	Stand on one foot with eyes closed/s	Balance beam/s
99	Male	45.02	2.50	2.89	33.2	60.42	1.72
	Female	38.84	2.00	3.03	31.0	51.95	1.81
95	Male	40.92	2.73	3.40	29.51	58.49	2.16
	Female	31.04	2.38	3.48	27.64	47.37	2.26
90	Male	36.17	2.93	3.86	26.31	53.21	2.37
	Female	25.96	2.88	3.81	24.95	43.17	2.41
85	Male	32.07	3.07	4.10	23.19	50.05	2.60
	Female	23.58	3.05	4.08	21.63	36.28	2.65
80	Male	29.33	3.25	4.27	21.33	46.55	2.80
	Female	21.51	3.15	4.22	19.27	31.00	2.75
75	Male	27.94	3.52	4.48	19.36	39.92	3.02
	Female	20.12	3.29	4.44	17.14	28.44	2.85
70	Male	25.67	3.70	4.60	17.35	36.00	3.20
	Female	19.22	3.47	4.65	15.69	25.95	2.98
65	Male	23.34	3.82	4.87	16.59	33.08	3.32
	Female	18.10	3.56	4.80	15.16	24.13	3.11
60	Male	21.63	3.96	5.09	15.9	32.18	3.42
	Female	16.95	3.79	4.96	14.0	22.29	3.29

#### 2.4.2 Children developmental behavior measures

The developmental behavior level of preschool children was evaluated with the Developmental Scale for Children aged 0–6 years (DSC), which has been recognized and applied in China (18, 19). This scale has been promulgated by the National Health Commission of the People’s Republic of China, which made it more authoritative and credible. The scale comprised a total of 261 indicators, which could evaluate the five attributes: gross motor, fine motor, language, adaptability, and social behavior in young children aged 1–84 months. Of these, there were 8–10 test items in each month age group. Notably, young children of different months of age had various test contents across each of the five attributes (see Supplementary Appendix 2). All tests were conducted using the specific inspection tools that matched the DSC. The specific tests, directions, materials and site layout, cautions and scoring criteria of the developmental behavior level of children test were based on the DSC. In this study, the results of each attribute’s score were represented by development quotient (DQ), and the DQ for each attribute and the total DQ could be calculated by the formula: DQ = (Mental Age/Actual Age) *100.

### 2.5 Statistical analysis

Statistical analysis was performed using SPSS 21.0 for Windows (SPSS). Normality was tested by means of the Kolmogorov–Smirnov test. All variables, except score of balance ability, were normally distributed (p>0.05). Therefore, one-way analysis of variance or Kruskal-Wallis test was used to compare the developmental behavior scores or balance ability scores of the four groups (TG, FG, SIG, CG) between pre- and post-intervention. Post-hoc analysis was conducted using the Bonferroni test. Effect size was evaluated with η^2^ (Eta partial squared) where 0.01 ≤ η^2^ < 0.06 constitutes a small effect, 0.06 ≤ η^2^ < 0.14 a medium effect, and η^2^ ≥ 0.14 a large effect (35). Moreover, a paired-sample t-test or non-parametric paired Wilcoxon signed-rank test was performed to compare the developmental behavior scores or balance ability scores of the four groups before and after the intervention. Cohen’s d effect size (ES) was used to quantify the magnitude of the training effect. The level of ES was defined as trivial (0.0–0.2), small (0.2–0.6), moderate (0.6–1.2), large (1.2–2.0), and very large (>2.0) (36). The Spearman correlation analysis was used to examine the correlations between developmental behavior scores and balance ability scores. For normal distributed variable, results were expressed as Mean ± SD, while for abnormal distributed indices, data were expressed as Median (Quartile). The significance level for all statistical analyses was set at *p* < 0.05.

## 3 Results

### 3.1 Scores from developmental scale for children

**Figure 2:**
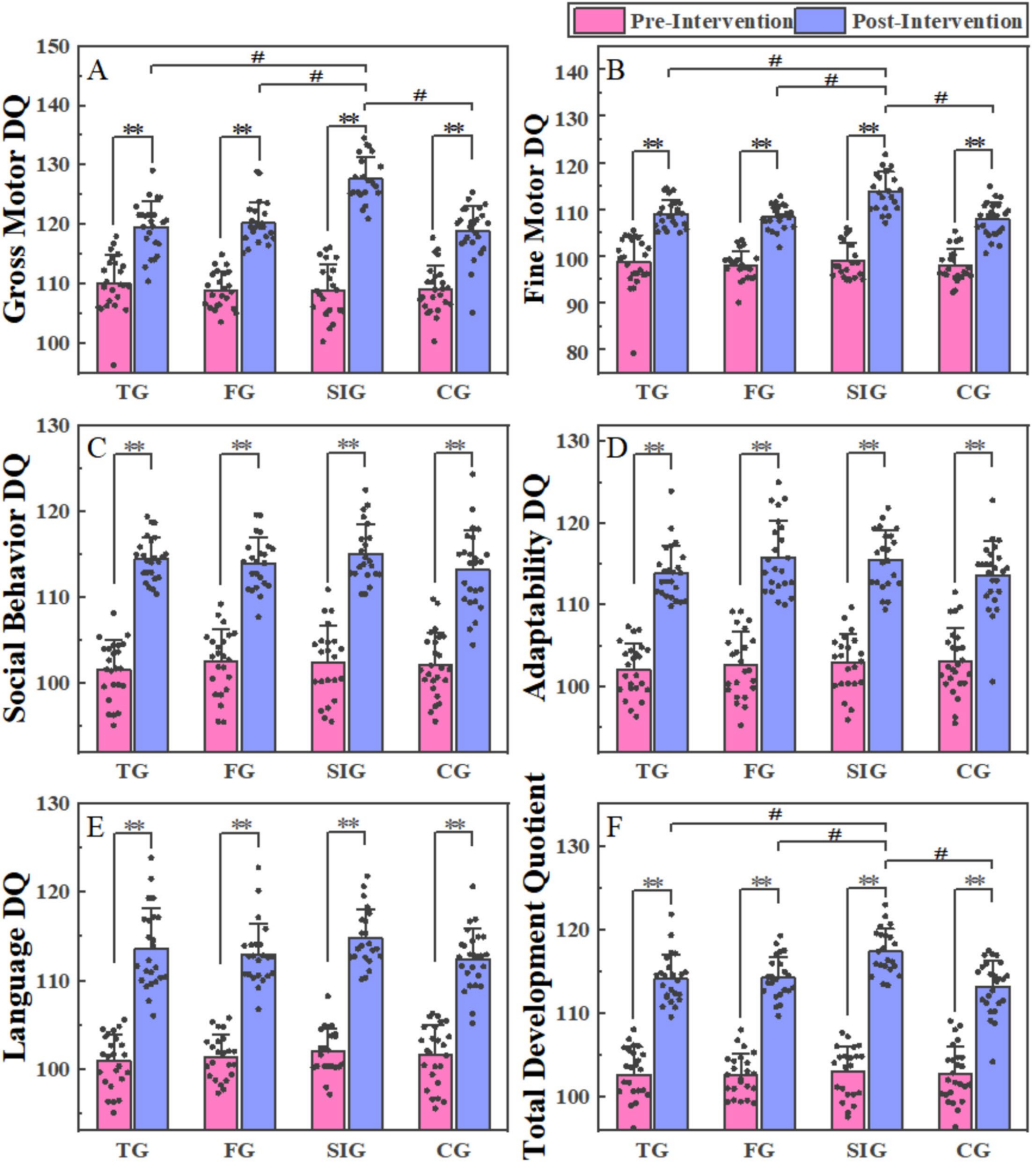
The comparison of the results of developmental behavior indicators (GMDQ, FMDQ, LADQ, ADDQ, SBDQ, and TDQ) before and after four different physical education curriculum interventions (CG, FG, SIG, and CG).

Figure 2 represents the comparison of the results of developmental behavior indicators (GMDQ, FMDQ, LADQ, ADDQ, SBDQ, and TDQ) before and after four different physical education curriculum interventions (CG, FG, SIG, and CG). There was no significant difference in all developmental behavior indicators among the four groups before the intervention. However, significant differences were observed in the gross motor development quotient, fine motor development quotient, and total development quotient among groups after the intervention (GMDQ: *F* = 23.057, *p* = 0.000, eta-square = 0.435; FMDQ: *F* = 15.603, *p* = 0.000, eta-square = 0.342; TDQ: *F* = 9.554, *p* = 0.000, eta-square = 0.242). For the developmental behavior indicators of GMDQ, FMDQ and TDQ, SIG showed a higher score than TG (GMDQ: *p* = 0.000; FMDQ: *p* = 0.000; TDQ: *p* = 0.001), FG (GMDQ: *p* = 0.000; FMDQ: *p* = 0.000; TDQ: *p* = 0.002) and CG (GMDQ: *p* = 0.000; FMDQ: *p* = 0.000; TDQ: *p* = 0.000). The values of all developmental behavior indicators before and after four different physical education curriculum interventions are shown in Table 3.

**Table 3:** The values of developmental behavior indicators before and after four different physical education curriculum interventions.

	Gross motor DQ		Fine motor DQ		Adaptability DQ		Language DQ		Social behavior DQ		Total DQ	
	Pre-	Post-	Pre-	Post-	Pre-	Post-	Pre-	Post-	Pre-	Post-	Pre-	Post-
TG	110.17 ± 4.71	119.63 ± 4.36	98.80 ± 5.73	109.11 ± 2.99	102.09 ± 3.20	113.91 ± 3.34	100.89 ± 3.04	113.62 ± 4.58	101.57 ± 3.42	114.45 ± 2.53	102.70 ± 3.33	114.14 ± 2.88
FG	108.97 ± 3.08	120.27 ± 3.55	98.07 ± 3.07	108.43 ± 2.60	102.67 ± 4.02	115.89 ± 4.51	101.39 ± 2.51	112.98 ± 3.53	102.48 ± 3.76	113.91 ± 3.03	102.72 ± 2.51	114.29 ± 2.51
SIG	108.78 ± 4.62	127.68 ± 3.76	99.06 ± 3.89	113.99 ± 4.13	102.90 ± 3.57	115.58 ± 3.61	102.05 ± 2.61	114.83 ± 3.25	102.42 ± 4.28	115.07 ± 3.41	103.04 ± 3.06	117.43 ± 2.69
CG	109.15 ± 4.02	118.88 ± 4.37	98.08 ± 3.54	107.89 ± 3.61	103.15 ± 4.07	113.63 ± 4.18	101.67 ± 3.38	112.48 ± 3.42	102.12 ± 3.72	113.23 ± 4.60	102.84 ± 3.30	113.22 ± 3.17

DQ is short for Development Quotient. TG is short for Tennis Group, FG is short for Football Group, SIG is short for Sensory Integration Group, and CG is short for Control Group. Pre- is for the short of Pre-intervention, Post- is for the short of Post-intervention. Data are represented as Mean ± SD.

For the tennis group, there was a statistically significant increase in FMDQ, FMDQ, ADDQ, LADQ, SBDQ and TDQ in the post-intervention period compared to the baseline period (GMDQ: *p* = 0.000, Cohen’d = 1.700; FMDQ: *p* = 0.000, Cohen’d = 1.594; ADDQ: *p* = 0.000, Cohen’d = 3.207; LADQ: *p* = 0.000, Cohen’d = 2.497; SBDQ: *p* = 0.000, Cohen’d = 3.575; TDQ: *p* = 0.000, Cohen’d = 3.078). For the football group, there was a statistically significant increase in FMDQ, FMDQ, ADDQ, LADQ, SBDQ and TDQ in the post-intervention period compared to the baseline period (GMDQ: *p* = 0.000, Cohen’d = 2.639; FMDQ: *p* = 0.000, Cohen’d = 2.714; ADDQ: *p* = 0.000, Cohen’d = 2.430; LADQ: *p* = 0.000, Cohen’d = 2.911; SBDQ: *p* = 0.000, Cohen’d = 2.451; TDQ: p = 0.000, Cohen’d = 3.537). For the sensory integration group, there was a statistically significant increase in FMDQ, FMDQ, ADDQ, LADQ, SBDQ and TDQ in the post-intervention period compared to the baseline period (GMDQ: *p* = 0.000, Cohen’d = 3.280; FMDQ: *p* = 0.000, Cohen’d = 3.121; ADDQ: *p* = 0.000, Cohen’d = 3.674; LADQ: *p* = 0.000, Cohen’d = 4.546; SBDQ: *p* = 0.000, Cohen’d = 2.331; TDQ: *p* = 0.000, Cohen’d = 5.377). For the control group, there was a statistically significant increase in FMDQ, FMDQ, ADDQ, LADQ, SBDQ and TDQ in the post-intervention period compared to the baseline period (GMDQ: *p* = 0.000, Cohen’d = 1.879; FMDQ: *p* = 0.000, Cohen’d = 2.269; ADDQ: *p* = 0.000, Cohen’d = 1.836; LADQ: *p* = 0.000, Cohen’d = 2.618; SBDQ: p = 0.000, Cohen’d = 2.010; TDQ: *p* = 0.000, Cohen’d = 2.826).

### 3.2 Scores from balance ability test

**Figure 3:**
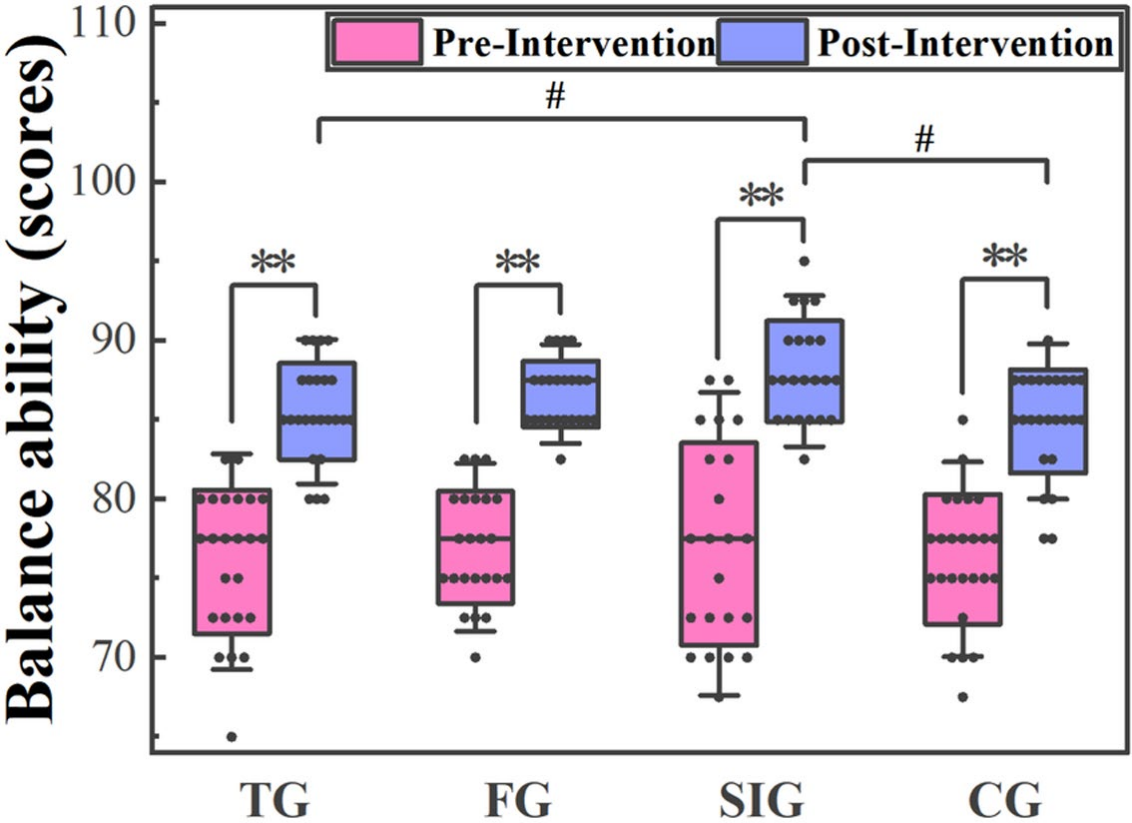
The comparison of balance ability scores before and after four different physical education curriculum interventions. Data were represented as Median (Quartile). Significant differences are indicated by asterisks (*p* < 0.05), double asterisks (*p* < 0.01) or # (*p* < 0.01). Noted that the points represent each subjects’ score and the black line was the median number.

Figure 3 illustrates the comparison of balance ability scores before and after four different physical education curriculum interventions. There was no significant difference in balance ability scores among the four groups before the intervention. However, significant differences were observed in balance ability scores among the four groups after the intervention (*p* = 0.015). Specifically, the SIG showed higher balance ability scores than TG (*p* = 0.011) and CG (*p* = 0.003), whereas no significant differences were identified between FG and SIG (*p* > 0.05).

For the tennis group, the balance ability scores before and after intervention were 77.50 (72.50, 80.00) and 85.00 (85.00, 87.50), respectively. There was a statistically significant increase in the scores in the post-intervention period compared to the baseline period (Z = -4.332, *p* = 0.000). For the football group, the balance ability scores before and after intervention were 77.50 (75.00, 80.00) and 87.50 (85.00, 87.50), respectively. There was a statistically significant increase in the scores in the post-intervention period compared to the baseline period (*Z* = −4.234, *p* = 0.000). For the sensory integration group, the balance ability scores before and after intervention were 77.50 (71.88, 83.13) and 87.50 (85.00, 90.00), respectively. There was a statistically significant increase in the scores in the post-intervention period compared to the baseline period (*Z* = −4.136, *p* = 0.000). For the control group, the balance ability scores before and after intervention were 77.50 (75.00, 78.75) and 85.00 (83.75, 87.50), respectively. There was a statistically significant increase in the scores in the post-intervention period compared to the baseline period (*Z* = −4.417, *p* = 0.000).

### 3.3 Correlation

Table 4 shows the correlations between developmental behavior indicators and balance ability among the four different physical education curriculum interventions before and after intervention. Before the intervention, there was a significant positive correlation between the balance ability of the four groups and their gross motor development quotient, fine motor development quotient, and total development quotient (GMDQ: TG: *r* = 0.864, *p* < 0.01; FG: *r* = 0.893, *p* < 0.01; SIG: *r* = 0.840, *p* < 0.01; CG: *r* = 0.862, *p* < 0.01; FMDQ: TG: *r* = 0.548, *p* < 0.01; FG: *r* = 0.681, *p* < 0.01; SIG: *r* = 0.488, *p* < 0.05; CG: *r* = 0.705, *p* < 0.01; TDQ: TG: *r* = 0.726, *p* < 0.01; FG: *r* = 0.799, *p* < 0.01; SIG: *r* = 0.613, *p* < 0.01; CG: *r* = 0.734, *p* < 0.01). After the intervention, significant positive correlation was also found between the gross motor development quotient, fine motor development quotient, total developmental quotient and balance ability in the four groups (GMDQ: TG: *r* = 0.743, *p* < 0.01; FG: *r* = 0.562, *p* < 0.01; SIG: *r* = 0.748, *p* < 0.01; CG: *r* = 0.694, *p* < 0.01; FMDQ: TG: *r* = 0.533, *p* < 0.01; FG: *r* = 0.454, *p* < 0.05; SIG: *r* = 0.772, *p* < 0.01; CG: *r* = 0.457, *p* < 0.05; TDQ: TG: *r* = 0.602, *p* < 0.01; FG: *r* = 0.502, *p* < 0.05; SIG: *r* = 0.781, *p* < 0.01; CG: *r* = 0.584, *p* < 0.01). In addition, a significant positive correlation between the balance ability of sensory integration group and its adaptability development quotient and social behavior development quotient was observed after the intervention (ADDQ: *r* = 0.678, *p* < 0.01; SB: *r* = 0.613, *p* < 0.01).

**Table 4:** The correlations between developmental behavior indicators and balance ability among the four different physical education curriculum interventions before and after intervention.

	Gross motor DQ		Fine motor DQ		Adaptability DQ		Language DQ		Social behavior DQ		Total DQ	
	Pre-	Post-	Pre-	Post-	Pre-	Post-	Pre-	Post-	Pre-	Post-	Pre-	Post-
TG	0.864**	0.743**	0.548**	0.533**	0.295	0.387	0.398	0.248	0.404	0.251	0.726**	0.602**
FG	0.893**	0.562**	0.681**	0.454*	0.289	0.238	0.346	0.366	0.321	0.345	0.799**	0.502*
SIG	0.840**	0.748**	0.488 *	0.772**	0.216	0.678**	0.283	0.207	0.307	0.613 **	0.613**	0.781**
CG	0.862**	0.694**	0.705**	0.457*	0.355	0.374	0.370	0.212	0.241	0.304	0.734**	0.584**

DQ is short for Development Quotient. TG, FG, SIG, and CG is short for Tennis Group, Football Group, Sensory Integration Group, and Control Group, respectively. Pre- is for the short of Pre-intervention, Post- is for the short of Post-intervention. Significant differences are indicated by asterisks (*p* < 0.05) or double asterisks (*p* < 0.01).

## 4 Discussion

This study presented some of the first findings on the effects of four different structured physical education curriculum interventions on developmental behavior and balance ability of preschool children aged 4–6 years old and their correlation. It was interestingly found that all interventions result in greater improvements in total development quotient (DQ), gross motor DQ, fine motor DQ, language DQ, adaptability DQ, social behavior DQ, and balance ability after the intervention compared to baseline. Furthermore, the sensory integration group showed greater improvements in GMDQ, FMDQ, TDQ and balance ability than the other three groups after the intervention. However, one special finding of interest was that no significant difference in balance ability between football group and sensory integration group. This partially supported our hypothesis. Moreover, we also found a positive and significant correlations between ADDQ, SBDQ and balance ability after sensory integration intervention. To our knowledge, this is the first study to simultaneously compare the effects of four different physical education curriculum interventions on developmental behavior and balance ability, and the correlation between these two factors in preschool children.

The traditional physical education curriculum in kindergartens has been suggested as a primary way to promote the development of motor skills and physical health of preschool children. However, there was little robust evidence on the wildly use of curriculum in kindergartens that simultaneously embody the characteristics of interest, structure and sports-led (8, 37). In this study, the primary characteristics of different curriculum interventions were football curriculum intervention dominated by lower limbs, tennis curriculum intervention dominated by upper limbs and sensory integration curriculum intervention dominated by the whole body coordination. However, both the control group and these three intervention groups showed significant increases in developmental behavior and balance ability after the 12-week intervention compared to their respective baselines. The results were similar to the published studies (2, 4, 26). For example, in a recent study, Li et al. (26) found that martial arts sensory teaching group and martial arts traditional teaching group had the same effect on improving the total score of motor skills. However, in many studies, there was no significant change after the intervention of traditional physical education curriculum (2, 4, 7, 38). Compared to relevant previous researches, it seemed that the results of consistent changes in the traditional physical education curriculum would be related to the curriculum design of this study, the result of the almost consistent volume, duration and intensity of physical activity in the curriculum, as well as the engagement of the same muscle groups.

To date, no study has concurrently compared the effects of the aforementioned four physical education curriculum interventions on the developmental behavior of preschool children (7, 9, 39). For example, Luka et al. (39) merely conducted a comparison between the regular tennis training and the exergame plus regular tennis training in gross motor development. Other studies have compared sensory integration training interventions with blank controls (7). In this study, after intervention, the sensory integration group exhibited significantly higher scores on the GMDQ, FMDQ, and TDQ than the tennis, football and control groups, while the football group, tennis group and control group had no significant differences in all developmental behavior indicators. Compared with tennis courses dominated by upper limbs and football courses dominated by lower limbs, sensory integration courses dominated by whole body coordination are more systematic and coordinated. Previous studies argued that the sensory integration training was a process that involves the integration of multiple sensory inputs, including vision, audition, vestibular sensation, proprioception, tactile, and olfactory information (7, 40). The locomotor and object control of gross motor skills are correlated with vestibular function and proprioception in sensory integration (4, 23). Therefore, this may explain why the GMDQ, FMDQ, and TDQ exhibited better outcomes following the sensory integration curriculum intervention. Moreover, the similar curriculum structure, content, exercise duration, volume, and intensity across the tennis, football, and control groups could potentially account for the absence of significant differences among them.

In previous studies, gross motor and fine motor skills have generally been used to evaluate the impact of different interventions, ages, relative ages, and genders on the developmental features, motor competence, activity levels, and motor skills among both healthy and sick young children. These studies have consistently reported positive effects (2, 12–16). It was found that the Test of Gross Motor Development-Second Edition (TGMD-2), TGMD-3, Movement Assessment Battery for Children-2, Bayley Scale of Infant Development, Gesell Development Scale, Cognitive Assessment System, Accelerometer and other tools were widely used to access gross motor skills, fine motor skills, physical activity, cognition and so on. These assessments were extensively employed in the research field of young children developmental behavior (2, 12, 41–43). However, in this study, we employed the Developmental Scale for Children aged 0–6 years (DSC), a tool specifically adapted for Chinese children, which assesses language, adaptability, and social behavior, as well as gross motor and fine motor skills (18, 19). A Chinese study has found that both parent-led family support training and non-parent-led family support training significantly increased the TDQ in children with psychomotor retardation. The parent-led family support training group showed significant improvements in gross motor, fine motor, and adaptability post-intervention compared to the non-parent-led family support training group (44). This partially supports the results of the present study. To our knowledge, this is the first study to use the DSC to explore the effects of different curriculum interventions on young children’s developmental behavior.

In terms of balance ability, it has been suggested that the human balance system may develop with age, of which children aged 3–6 years are particularly sensitive to the development of their balance ability, while the balance regulation mechanism in children aged 7 years is similar to that of adults (12). In this study, all participants were preschool children who were in a particularly sensitive phase of balance ability development. Each of the four physical education curriculum interventions involved lower limb movements and balance control, while maintaining a consistent volume and intensity of physical activity in the curriculum. These may be why all four physical education curricula have a significant effect in promoting balance ability. Some studies argued that children’s balance depended on interaction with surrounding environments and exercises of muscle during the growth process (12, 45). In the present study, there was no significant difference in balance ability between football group and sensory integration group after intervention, which may be attributed to the dominance of lower limb muscles in the overall movement patterns of football.

Some studies argued that motor competence was defined as gross motor skill competency, while the gross motor skill competence was defined as proficiency in a range of fundamental movements skills (13, 29, 46). The fundamental movement skills were often described more precisely as basic stability (e.g., static balance and dynamic balance), object control (manipulation) and locomotor movements (3, 24, 46). These points seem to indicate that there was a positive relationship between balance ability and gross motor and fine motor. Furthermore, it was believed that the essence of sensory integration training was to use gross motor activities to activate the vestibular and somatosensory systems (47). In this study, positive correlations were observed between the GMDQ, FMDQ, TDQ, and balance ability at both baseline and post- intervention. These findings were consistent with the general conclusion of the study (13, 46, 47). Moreover, this study was the first to find a significant positive correlation between balance ability and SBDQ and ADDQ after intervention. It has been suggested that the essence of sensory integration training was employing play activities and sensory-enhanced interactions to elicit the child’s adaptive response (48). The goal of sensory integration training was to increase the child’s ability to integrate sensory information, thereby demonstrating more organized and adaptive behaviors, including social skill, motor planning, and perceptual skill (48, 49). Therefore, this might be the reason for the correlation between balance ability and SBDQ and ADDQ after intervention.

Exercise has positive effects on young children’s physical and brain function (40, 50). In this study, the exercise content of all four curriculum interventions involved the coordination of whole-body muscles and the control of small muscle groups. This helps young children to control their body, maintain balance and enhance the fine control of local muscles, thereby improving gross motor, fine motor and balance ability. Instruction comprehension, mutual communication and cooperation during exercise may potentially improve language and social behavior ability. Moreover, young children require to adapt to changing environments and various situations in physical activity during exercise, which may potentially enhance adaptability. Previous studies argued that the brain regulates motor behavior and performance while motor training influences brain function and structure (50). Long-term exercise stimulates the development of the nervous system, enhances neural pathways, improves the plasticity of the brain and nerves, and thus promotes the growth and development of young children (40). In addition, the better results of the sensory integration curriculum intervention could potentially be attributed to the mechanism that vestibular and proprioceptive input can modulate the processing of sensory information in the reticular formation and limbic system and achieve the ideal level of alertness in the central nervous system (7, 51, 52).

However, it should be acknowledged that this study has several limitations. Firstly, the sample size of the current study was relatively small, which may influence the credibility of the current research. A larger sample size would be effective to acquire a greater generalizability. Secondly, owing to unpredictable and uncontrollable factors, the number of participants included in this study did not reach the optimal sample size as calculated by G*power. Increasing the number of additional participants could potentially address this issue. Thirdly, the control measures implemented in China during the post-epidemic period could potentially influence the daily activities of participants and the efficacy of the intervention, thereby introducing the risk of interference from other irrelevant variables. If possible, future experiments’ recruitment and intervention implementation strategies should avoid such period. Lastly, the scales and test contents used in the current study originated from China and were more applicable to Chinese preschool children, which may limit the universal applicability of the research results. Future studies should consider adopting internationally recognized scales to evaluate the developmental behavior of preschool children and employing more precise and advanced laboratory equipment to assess balance ability, so as to improve the accuracy and credibility of research results. Moreover, it is a difficult task to intervene and test preschool children, and there would be many emergencies in the process of intervention that would affect the effect of a single intervention. A more robust study design with strict execution would be needed to solve the issue in the future study. Furthermore, future research could build upon the current findings to perform comparative studies across various ages and genders. This approach could potentially be more effective in enhancing the motor skills of young children and improving the quality of physical education curriculum.

## 5 Conclusion

In conclusion, all four different physical education curriculum interventions significantly improved developmental behavior and balance ability. Among them, the sensory integration physical education curriculum intervention has been found to be the most effective in improving GMDQ, FMDQ, TDQ and balance ability in preschool children. There was significant positive correlation between balance ability and GMDQ, FMDQ, and TDQ before and after the intervention. Notably, a positive and significant correlations between ADDQ, SBDQ, and balance ability after sensory integration intervention was found. This finding suggested that sensory integration training should be given priority in the future routine physical education curriculum design, so as to promote the efficient improvement of preschool children’s developmental behavior and motor competence.

## Data Availability

The raw data supporting the conclusions of this article will be made available by the authors, without undue reservation.

## References

[1] Van Hooren B De Ste CM. Sensitive periods to train general motor abilities in children and adolescents: do they exist? A critical appraisal Strength. Cond J. (2020) 42:7–14. doi: 10.1519/SSC.0000000000000545

[2] Zeng N Ayyub M Sun H Wen X Xiang P Gao Z. Effects of physical activity on motor skills and cognitive development in early childhood: a systematic review. Biomed Res Int. (2017) 2017:1–13. doi: 10.1155/2017/2760716, PMID: 29387718 PMC5745693

[3] Laurent CW Burkart S Andre C Spencer RMC. Physical activity, fitness, school readiness, and cognition in early childhood: a systematic review. J Phys Act Health. (2021) 18:1004–13. doi: 10.1123/jpah.2020-0844, PMID: 34140418 PMC9297301

[4] Fu T Zhang D Wang W Geng H Lv Y Shen R. Functional training focused on motor development enhances gross motor, physical fitness, and sensory integration in 5–6-year-old healthy Chinese children. Front Pediatr. (2022) 10:10. doi: 10.3389/fped.2022.936799, PMID: 35899135 PMC9309543

[5] Goldfield GS Harvey A Grattan K Adamo KB. Physical activity promotion in the preschool years: a critical period to intervene. Int J Env Res Pub He. (2012) 9:1326–42. doi: 10.3390/ijerph9041326, PMID: 22690196 PMC3366614

[6] Ministry of education of the people’s republic of china. National educational development bulletin. (2023). Available at: http://www.moe.gov.cn/jyb_sjzl/sjzl_fztjgb/202307/t20230705_1067278.html (Accessed March 1, 2024).

[7] Lin CL Min YF Chou LW Lin CK. Effectiveness of sensory processing strategies on activity level in inclusive preschool classrooms. Neuropsych Dis Treat. (2012) 8:475–81. doi: 10.2147/NDT.S37146, PMID: 23118541 PMC3484897

[8] Healey D Milne B Healey M. Adaption and implementation of the engage programme within the early childhood curriculum. Sci Rep. (2022) 12:21580. doi: 10.1038/s41598-022-25655-8, PMID: 36517624 PMC9751130

[9] Wang G Zi Y Li B Su S Sun L Wang F. The effect of physical exercise on fundamental movement skills and physical fitness among preschool children: study protocol for a cluster-randomized controlled trial. Int J Env Res Pub He. (2022) 19:6331. doi: 10.3390/ijerph19106331, PMID: 35627867 PMC9141773

[10] Jones D Innerd A Giles EL Azevedo LB. Association between fundamental motor skills and physical activity in the early years: a systematic review and meta-analysis. J Sport Health Sci. (2020) 9:542–52. doi: 10.1016/j.jshs.2020.03.001, PMID: 33308805 PMC7749255

[11] Kuzik N Poitras VJ Tremblay MS Lee E Hunter S Carson V. Systematic review of the relationships between combinations of movement behaviours and health indicators in the early years (0-4 years). *Bmc*. Public Health. (2017) 17:17. doi: 10.1186/s12889-017-4851-1, PMID: 29219071 PMC5773877

[12] Jiang GP Jiao XB Wu SK Ji ZQ Liu WT Chen X. Balance, proprioception, and gross motor development of Chinese children aged 3 to 6 years. J Motor Behav. (2018) 50:343–52. doi: 10.1080/00222895.2017.1363694, PMID: 28915098

[13] Barnett LM Lai SK Veldman SLC Hardy LL Cliff DP Morgan PJ. Correlates of gross motor competence in children and adolescents: a systematic review and Meta-analysis. Sports Med. (2016) 46:1663–88. doi: 10.1007/s40279-016-0495-z, PMID: 26894274 PMC5055571

[14] Navarro-Patón R Arufe-Giráldez V Sanmiguel-Rodríguez A Mecías-Calvo M. Differences on motor competence in 4-year-old boys and girls regarding the quarter of birth: is there a relative age effect? Children. (2021) 8:141. doi: 10.3390/children8020141, PMID: 33668429 PMC7917671

[15] Rao N Chan S Su Y Mirpuri S Richards B Sun J. Early motor development in China: secular trends among 4-year-olds. Early Child Dev Care. (2023) 193:95–108. doi: 10.1080/03004430.2022.2064460

[16] Navarro-Patón R Mecías-Calvo M Rodríguez Fernández JE Arufe-Giráldez V. Relative age effect on motor competence in children aged 4–5 years. Children. (2021) 8:115. doi: 10.3390/children8020115, PMID: 33561982 PMC7914921

[17] Chen S Zhao J Hu X Tang L Li J Wu D. Children neuropsychological and behavioral scale-revision 2016 in the early detection of autism spectrum disorder. Front Psych. (2022) 13:893226. doi: 10.3389/fpsyt.2022.893226, PMID: 35935438 PMC9354041

[18] National Health Commission of the People’s Republic of China. Developmental scale for children aged 0–6 years. (2017). Available at: http://www.nhc.gov.cn/ewebeditor/uploadfile/2017/10/20171026154358287.pdf. (Accessed March 1, 2024).

[19] Zheng X Li R Wang L Yang H Li L Cui J. The association of cesarean section with overweight and neurodevelopment of Chinese children aged 1–5 months. Front Pediatr. (2022) 10:940422. doi: 10.3389/fped.2022.940422, PMID: 36081630 PMC9445438

[20] Li H Feng J Wang B Zhang Y Wang C Jia F. Comparison of the children neuropsychological and behavior scale and the griffiths mental development scales when assessing the development of children with autism. Psychol Res Behav Ma. (2019) 12:973–81. doi: 10.2147/PRBM.S225904, PMID: 31802957 PMC6801569

[21] Blodgett JM Cooper R Pinto PS Hamer M. Stability of balance performance from childhood to midlife. Pediatrics. (2022) 150:150. doi: 10.1542/peds.2021-055861, PMID: 35670126

[22] Yanovich E Bar-Shalom S. Static and dynamic balance indices among kindergarten children: a short-term intervention program during COVID-19 lockdowns. Children. (2022) 9:939. doi: 10.3390/children9070939, PMID: 35883923 PMC9319221

[23] Mache MA Todd TA. Gross motor skills are related to postural stability and age in children with autism spectrum disorder. Res Autism Spect Dis. (2016) 23:179–87. doi: 10.1016/j.rasd.2016.01.001

[24] Bolger LE Bolger LA O’Neill C Coughlan E O’Brien W Lacey S. Global levels of fundamental motor skills in children: a systematic review. J Sport Sci. (2021) 39:717–53. doi: 10.1080/02640414.2020.1841405, PMID: 33377417

[25] Mickle KJ Munro BJ Steele JR. Gender and age affect balance performance in primary school-aged children. J Sci Med Sport. (2011) 14:243–8. doi: 10.1016/j.jsams.2010.11.002, PMID: 21276751

[26] Li B Li R Qin H Chen T Sun J. Effects of Chinese martial arts on motor skills in children between 5 and 6 years of age: a randomized controlled trial. Int J Env Res Pub He. (2022) 19:10204. doi: 10.3390/ijerph191610204, PMID: 36011834 PMC9408615

[27] Fong SSM Tsang WWN Ng GYF. Taekwondo training improves sensory organization and balance control in children with developmental coordination disorder: a randomized controlled trial. Res Dev Disabil. (2012) 33:85–95. doi: 10.1016/j.ridd.2011.08.023, PMID: 22093652

[28] Anderson N Button C Lamb P. The effect of educational gymnastics on postural control of young children. Front Psychol. (2022) 13:13. doi: 10.3389/fpsyg.2022.936680, PMID: 36033080 PMC9399810

[29] Baumeister D Peters E Pruessner J Howes O Chadwick P. The effects of voice content on stress reactivity: a simulation paradigm of auditory verbal hallucinations. Schizophr Res. (2022) 243:225–31. doi: 10.1016/j.schres.2019.07.019, PMID: 31377050 PMC9205337

[30] Alpert B Field T Goldstein S Perry S. Aerobics enhances cardiovascular fitness and agility in preschoolers. Health Psychol. (1990) 9:48–56. doi: 10.1037//0278-6133.9.1.48, PMID: 2323328

[31] Bi Z Yongfeng L Yan Z. Test and evaluation of balance ability of children aged 3~6 years old. Chin Sch Phys Educ. (2022) 41:65–7.

[32] Bi Z Mengning L Pengfei J Jianjin L. Research on the “Three-dimensional Movement” ability assessment system for children aged 3~6. Sports J. (2022) 29:131–7. doi: 10.16237/j.cnki.cn44-1404/g8.2022.04.008

[33] Ministry of education of the people’s republic of china. Early learning and development guidelines for children aged 3 to 6 years. (2012). Available at: https://www.unicef.cn/sites/unicef.org.china/files/2018-10/2012-national-early-learning-development-guidelines.pdf. (Accessed March 1, 2024).

[34] Ministry of education of the people’s republic of china. Guidelines for kindergarten education (trial). (2001). Available at: http://www.moe.gov.cn/srcsite/A06/s3327/200107/t20010702_81984.html (Accessed March 1, 2024).

[35] Ferraz RMP van den Tillaar R Pereira A Marques MC. The effect of fatigue and duration knowledge of exercise on kicking performance in soccer players. J Sport Health Sci. (2019) 8:567–73. doi: 10.1016/j.jshs.2016.02.001, PMID: 31720069 PMC6834994

[36] Wang L Qiao M Tao H Song X Shao Q Wang C. A comparison of muscle activation and concomitant intermuscular coupling of antagonist muscles among bench presses with different instability degrees in untrained men. Front Physiol. (2022) 13:13. doi: 10.3389/fphys.2022.940719, PMID: 36148298 PMC9486837

[37] Abusleme-Allimant R Hurtado-Almonacid J Reyes-Amigo T Yáñez-Sepúlveda R Cortés-Roco G Arroyo-Jofré P. Effects of structured and unstructured physical activity on gross motor skills in preschool students to promote sustainability in the physical education classroom. Sustain Basel. (2023) 15:10167. doi: 10.3390/su151310167

[38] Navarro-Patón R Martín-Ayala JL Martí González M Hernández A Mecías-Calvo M. Effect of a 6-week physical education intervention on motor competence in pre-school children with developmental coordination disorder. J Clin Med. (2021) 10:1936. doi: 10.3390/jcm10091936, PMID: 33946206 PMC8124766

[39] Šlosar L de Bruin ED Fontes EB Plevnik M Pisot R Simunic B. Additional exergames to regular tennis training improves cognitive-motor functions of children but may temporarily affect tennis technique: a single-blind randomized controlled trial. Front Psychol. (2021) 12:12. doi: 10.3389/fpsyg.2021.611382, PMID: 33790833 PMC8005621

[40] Lane SJ Mailloux Z Schoen S Bundy A May-Benson TA Parham LD. Neural foundations of ayres sensory integration®. Brain Sci. (2019) 9:153. doi: 10.3390/brainsci9070153, PMID: 31261689 PMC6680650

[41] Fisher A Boyle JM Paton JY Tomporowski P Watson C McColl JH. Effects of a physical education intervention on cognitive function in young children: randomized controlled pilot study. BMC Pediatr. (2011) 11:97. doi: 10.1186/1471-2431-11-97, PMID: 22034850 PMC3217848

[42] Nagy ÁV Wilhelm M Domokos M Győri F Berki T. Assessment tools measuring fundamental movement skills of primary school children: a narrative review in methodological perspective. Sports. (2023) 11:178. doi: 10.3390/sports11090178, PMID: 37755855 PMC10534471

[43] Logan SW Robinson LE Rudisill ME Wadsworth DD Morera M. The comparison of school-age children’s performance on two motor assessments: the test of gross motor development and the movement assessment battery for children. Phys Educ Sport Peda. (2014) 19:48–59. doi: 10.1080/17408989.2012.726979

[44] Yueyi Q Jia S Tian T Youfang H. Effects of family support training on the effectiveness of rehabilitation training for children with psychomotor developmental delay. Jiangsu Medicine. (2021) 47:886–9. doi: 10.19460/j.cnki.0253-3685.2021.09.006.

[45] Hsu Y Kuan C Young Y. Assessing the development of balance function in children using stabilometry. Int J Pediatr Otorhi. (2009) 73:737–40. doi: 10.1016/j.ijporl.2009.01.016, PMID: 19232750

[46] Barros WMA Silva KGD Silva RKP Souza APDS Silva ABJD Silva MRM. Effects of overweight/obesity on motor performance in children: a systematic review. Front Endocrinol. (2022) 12:12. doi: 10.3389/fendo.2021.759165, PMID: 35126307 PMC8812008

[47] Mailloux Z Roley SS. Sensory integration. Bethesda: AOTA Press (2010).

[48] Case-Smith J Weaver LL Fristad MA. A systematic review of sensory processing interventions for children with autism spectrum disorders. Autism. (2015) 19:133–48. doi: 10.1177/1362361313517762, PMID: 24477447

[49] Baranek G. Efficacy of sensory and motor interventions for children with autism. J Autism Dev Disord. (2002) 32:397–422. doi: 10.1023/A:1020541906063 12463517

[50] Li W Zhang Q Yang R Liu B Chen G Wang B. Characteristics of resting state functional connectivity of motor cortex of high fitness level college students: experimental evidence from functional near infrared spectroscopy (fNIRS). Brain Behav. (2023) 13:e3099. doi: 10.1002/brb3.3099, PMID: 37303301 PMC10338804

[51] Williams MS Shellenberger S. How does your engine run? A Leader’s guide to the alert program for self-regulation. Albuquerque: Therapy Works (1994).

[52] Bundy AC Lane SJ Murray EA. Sensory integration theory and practice. Philadelphia: Davis Company (2002).

