# Supplementary material for: A comparative study of four physical education curricula on the developmental behavior of Chinese preschool children aged 4 to 6 years and its correlation with balance ability
Source: Front Public Health. 2025 Mar 10;13:1477001. doi: 10.3389/fpubh.2025.1477001 (PMC11930833; doi:10.3389/fpubh.2025.1477001)
Supplement: Supplementary file 1 [file Data_Sheet_1.PDF]

Supplementary Appendix 1:

Description of the main content programme of the four interventions

Interventions	Phases	Themes	Contents
Tennis Group (TG)	Phase 1 Start Phase (1-4 Week)	Interest cultivating	**1. Perception related games (5 min):** Individuals free hand throw the tennis ball vertically; overhead ball toss for a high-five reception; free-hand tennis serves.<br>**2. Footstep related games (5 min):** "Happy animals" game: Imitate the footsteps of leopards, frogs, rabbits, ducks, snakes, spiders, and other small animals.<br>**3. Skill-related studying and games (10 min):** Learning and practicing tennis forehand movement; "Command - jump with both feet shoulder width apart" game; "Make an ice cream" game: Catch a tennis ball by a cone.
	Phase 2 Advance Phase (5-8 Week)	Motion perception enhancing	**1. Perception related games (5 min):** Pairs of students free hand throw and catch the tennis ball; bouncing ball catch; "Ant movement" game: Put tennis balls on a racket and then transport them to a designated location.<br>**2. Footstep related games (5 min):** "Happy animals" game: Imitate the footsteps of leopards, frogs, rabbits, ducks, snakes, spiders, and other small animals; "Through the tunnel" game: Students line up and roll a tennis ball through a tunnel formed by their legs.<br>**3. Skill-related studying and games (10 min):** Practicing tennis forehand movement with a plastic bag over the racket; "Command - Hold the racket and jump with both feet shoulder width apart" game; "Make an ice cream" game: Catch a tennis ball by a cone across the net; Stand on a fixed spot and turn sideways to hit the ball over the net with the racket.

	Phase 3 Improve Phase (9-12 Week)	Motor ability improving	**1. Perception related games (5 min):** “Ant movement” game: Put tennis balls on a racket and then transport them to a designated location; “Count the tennis ball bounces” game: Touch the ball vertically with the racket and record the number of bounces.<br>**2. Footstep related games (5 min):** “Through the tunnel” game: Students line up and roll a tennis ball through a tunnel formed by their legs; “Command - Execute the number shape” game: Follow the number in the command to run the corresponding shape.<br>**3. Skill-related studying and games (10 min):** Practicing tennis forehand movement with a plastic bag over the racket; Stand on a fixed spot and turn sideways to hit the ball over the net with the racket; “Upgrade version of making an ice cream” game: Catch a tennis ball by racket across the net.
Football group (FG)	Phase 1 Start Phase (1-4 Week)	Interest cultivating	**1. Perception related games (5 min):** Kick the ball lightly and tentatively with the back or top of your foot; “Step on the red mark” game: Put a red mark on the football and step it with one foot (the football stays in a fixed spot); “Upgrade version of stepping on the red mark” game: Put a red mark on the football and step it alternately with both feet (the football stays in a fixed spot).<br>**2. Footstep related games (5 min):** “Happy animals” game: Imitate the footsteps of crabs, leopards, frogs, rabbits, ducks, snakes, spiders, and other small animals.<br>**3. Skill-related studying and games (10 min):** Learning and practicing the inside-of the-foot kick movement; “Run on command game: Run to the sign in the direction of the command and stop”; “Stop in the sweet spot” game: After the run-up, the support foot falls in the circle.
	Phase 2 Advance Phase (5-8 Week)	Motion perception enhancing	**1. Perception related games (5 min):** Kick the ball lightly with the back or top of your foot; “Step on the red mark” game: Put a red mark on the football and step it with one foot (the football position is not fixed); “Shooting” game: Kick a stationary football into a homemade goal.<br>**2. Footstep related games (5 min):**

			“Happy animals’ game: Imitate the footsteps of crabs, leopards, frogs, rabbits, ducks, snakes, spiders, and other small animals”; “Conveyor belt” game: Move sideways around two designated marker buckets.<br>**3. Skill-related studying and games (10 min):** Practicing the inside-of the-foot kick movement; “Run in reverse on command” game: Run to the sign in the opposite direction of the command and stop; “Stop in the sweet spot” game: After the run-up, the support foot falls in the circle; “Look in the mirror” game: Swing your legs face to face with your partner.
	Phase 3 Improve Phase (9-12 Week)	Motor ability improving	**1. Perception related games (5 min):** Kick the ball with the back or top of your foot; “Upgrade version of stepping on the red mark” game: Put a red mark on the football and step it alternately with both feet (the position is not fixed); “Upgrade version of Shooting” game: Kick a stationary football to your partner.<br>**2. Footstep related games (5 min):** “Happy animals game: Imitate the footsteps of leopards, frogs, rabbits, ducks, snakes, spiders, and other small animals”; “Reverse password” game: Listen to the password and touch the marker in the opposite direction.<br>**3. Skill-related studying and games (10 min):** Practicing the inside-of the-foot kick movement; “Touch” game: Gently touch the ball with the inside of your foot and pass it to your partner at a fixed distance of 60 cm; “Accurate Delivery” game: Two partners face each other and kick the ball; “I am the champion” game: Use the inside-of the-foot kick movement to kick a football into the goal.
Sensory Integration Group (SIG)	All Phase (1-12 week)	Sensory Integration ability enhancing	**1. “Strength and endurance” game:** “Happy animals”: Imitate the footsteps of leopards, frogs, rabbits, ducks, snakes, spiders, and other small animals”; “Run along the line”: Run along the curve and perform the tasks marked on the curve, such as jump 3 times, crawl, and so on; “We are porters”: A group of students work together to drag ropes (tied with tires of different weights) to their destination.

2. “Balance and coordination” game: “Don't fall”: Draw two lines 15-20 cm apart on the ground and pass smoothly through the middle of the line; “We are wooden people”: Stand still on one foot or two feet with eyes open or closed on a balance pad; “We are robots”: Open or close your eyes on a balance mat and step in place.

3. “Fine motor” game: “Make the ball disappear”: Use chopsticks, spoons, pliers and other tools to clamp the ping-pong ball to the destination; “Catch the Plane”: Fold the paper plane, make the paper plane fly and chase it; “Find it, stick it”: Follow the prompts to find the sticker and stick the sticker in the designated location.

4. “body awareness” game: “Cohort exercise”: Listen to the command and turn in the direction of the command; “Let's imitate”: The teacher does the movements and the students imitate them; “Rhythm Master”: Perform rhythmic upper-limb or lower-limb exercises to rhythmic music.

5. “Vestibular stimulation” game: “Dizzy Frog”: Turn around many circles, then conduct a standing long jump; “Walk in a straight line”: Turn around many circles, then walk in the specified straight line as far as possible; “Reverse command”: Run in the opposite direction after hearing the command; “Throwing a beanbag”: Running around a fixed field and dodging the beanbag; “Touch objects on command”: When the command is given, the student runs quickly to touch the objects in the command.

6. “Multisensory coordination” game: “Music rhythm”: Combined with the music rhythm, students follow the beat to do a variety of actions, such as clapping, jumping, turning, etc; “Jump over the line”: Jump with left foot or right foot over the line; “Target Throw”: Set targets of different colors and shapes and have students drop a ball or frisbee into them.

7. “Interesting competition” game: “Orienteering”: The puzzle pieces are placed in a fixed position, and under the guidance of the teacher, the students find them and do the puzzle; “Obstacle Relay”:

			Students complete a relay race on a course that requires jumping, crawling, throwing and so on.
Control Group (CG)	All Phase (1-12 week)	traditional physical education curriculum	**1. Rhythmic exercises (3 min):** Standing rhythmic exercise, or stationary drill.<br>**2. Group games (6 min):** Slapping ball; Relay race; Single leg jump; Cart pushing run.<br>**3. Gymnastics (3 min):** Basic movement exercise; Cohort exercise; Calisthenics.<br>**4. Free play (8 min):** Unstructured free activities, Playground slides.
