# Supplementary material for: A comparative study of four physical education curricula on the developmental behavior of Chinese preschool children aged 4 to 6 years and its correlation with balance ability
Source: Front Public Health. 2025 Mar 10;13:1477001. doi: 10.3389/fpubh.2025.1477001 (PMC11930833; doi:10.3389/fpubh.2025.1477001)
Supplement: Supplementary file 2 [file Data_Sheet_2.PDF]

ICS 13.100
C 56

WS

中华人民共和国卫生行业标准

WS/T 580—2017

0岁～6岁儿童发育行为评估量表

Developmental scale for children aged 0-6 years

2017－10－12发布 2018－04－01实施

中华人民共和国国家卫生和计划生育委员会 发布

前　言

本标准按照GB/T 1.1—2009 给出的规则起草。

本标准起草单位：首都儿科研究所、北京妇幼保健院、中国科学院心理研究所。

本标准主要起草人：陈博文、金春华、李瑞莉、张悦、张丽丽、李娜、高振敏、张家健、王建红、王晓燕、政晓果、高海涛、宋文红、梁卫兰、张峰、施建农、金星明、杨玉凤、静进、梁爱民、王惠珊、麻宏伟、章岚、王惠梅、张唯敏、高雪婷、杜其云、杨瑞华、王贺茹、卢慧敏、许琪、杨慧敏、王利红、肖峰、刘馨、辛倩倩、尹德卢、殷涛。

0岁～6岁儿童发育行为评估量表

1 范围

本标准规定了0岁～6岁（未满7周岁）儿童发育行为评估量表的评估内容、测查方法、发育商参考范围以及量表的使用。

本标准适用于0岁～6岁(未满7周岁)儿童发育行为水平的评估,是评估儿童发育行为水平的诊断量表。

2 术语和定义

下列术语和定义适用于本文件。

2.1

能区 attribute

量表测定的领域，本量表包括大运动、精细动作、语言、适应能力和社会行为五个能区。其中大运动能区指身体的姿势、头的平衡，以及坐、爬、立、走、跑、跳的能力；精细动作能区指使用手指的能力；语言能区指理解语言和语言的表达能力；适应能力能区指儿童对其周围自然环境和社会需要作出反应和适应的能力；社会行为能区指对周围人们的交往能力和生活自理能力。

2.2

智力年龄 mental age；MA

智龄

心理年龄

反映儿童智力水平高低的指标。

注：在编制的量表中，按年龄分组编制测查项目，若被试者通过3岁的测查项目，就表示他使用该量表测查的智力年龄为3岁。

2.3

发育商 development quotient；DQ

用来衡量儿童心智发展水平的核心指标之一，是在大运动、精细动作、认知、情绪和社会性发展等方面对儿童发育情况进行衡量。计算见式（1）：

$$发育商=\frac{智龄}{实际年龄}\times 100 \qquad (1)$$

3 评估内容

包括大运动、精细动作、语言、适应能力和社会行为5个能区，用于测查儿童发育行为状况，评估其发育程度。每个月龄组8～10个测查项目，共计261个测查项目。见附录A。

4 测查方法

4.1 测查工具

4.1.1 评估量表

0岁～6岁儿童发育行为评估量表，见附录A。

4.1.2 辅助工具

主试者使用与测查量表配套的标准化测查工具箱，以及诊查床、围栏床、小桌、小椅、楼梯等测查工具。

4.2 测查程序

4.2.1 计算实际月龄

4.2.1.1 首先根据被试者的测查日期和出生日期计算出被试者是几岁几月零几日，再把岁和日换算为月，以月龄为单位，月龄保留一位小数。

4.2.1.2 日换算成月为 30 天=1.0 个月，岁换算成月为 1 岁=12.0 个月。

4.2.2 标记主测月龄

与实际月龄最接近的月龄段为主测月龄，在主测月龄前用△标记，主测月龄介于量表两个月龄段之间的，视较小月龄为主测月龄。早产儿也按照实际月龄进行标记，无需矫正月龄。

4.2.3 测查启动与结束

4.2.3.1 主测月龄为启动月龄，先测查主测月龄的项目，无论主测月龄的某一能区的项目是否通过，需分别向前和向后再测查 2 个月龄，共 5 个月龄的项目。

4.2.3.2 向前测查该能区的连续 2 个月龄的项目均通过，则该能区的向前测查结束；若该能区向前连续 2 个月龄的项目有任何一项未通过，需继续往前测查，直到该能区向前的连续 2 个月龄的项目均通过为止。

4.2.3.3 然后从主测月龄向后测连续 2 个月龄的项目，若向后测查的该能区的连续 2 个月龄的项目均不能通过，则该能区的向后测查结束；若该能区向后连续 2 个月龄的项目有任何一项通过，需继续往后测查，直到该能区向后的连续两个月龄的项目均不通过为止。

4.2.3.4 所有能区均应按照 4.2.3.1～4.2.3.3 的要求进行测查。

4.2.4 记录方式

测查通过的项目用○表示；不通过的项目用×表示。

4.3 操作方法和测查通过要求

量表的操作方法和测查通过要求见附录B。

4.4 结果计算

4.4.1 各能区计分

4.4.1.1 1月龄～12月龄

每个能区1.0分，若只有一个测查项目，则该测查项目为1.0分；若有两个测查项目则各为0.5分。

4.4.1.2 15月龄～36月龄

每个能区3.0分，若只有一个测查项目，则该测查项目为3.0分；若有两个测查项目则各为1.5分。

4.4.1.3 42月龄～84 月龄

每个能区6.0分，若只有一个测查项目，则该测查项目为6.0分；若有两个测查项目则各为3.0分。

4.4.2 计算智龄

4.4.2.1 把连续通过的测查项目读至最高分（连续两个月龄通过则不再往前继续测，默认前面的全部通过），不通过的项目不计算，通过的项目（含默认通过的项目）分数逐项加上，为该能区的智龄。

4.4.2.2 将五个能区所得分数相加，再除以5就是总的智龄，保留一位小数。

4.4.3 计算发育商

发育商计算方法见式（1）。

5 发育商参考范围

发育商参考范围：＞130为优秀；110～129为良好；80～109为中等；70～79为临界偏低；＜70为智力发育障碍。

6 量表的使用

6.1 测查要求

6.1.1 测查环境应安静，光线明亮，4岁以下儿童允许一位家长陪伴,4岁及以上的儿童如伴有发育落后、沟通不利或者测查不配合的情况可有家长陪同。

6.1.2 主试者应严格按照操作方法和测查通过要求进行操作，避免被试儿童家长暗示、启发、诱导。

6.1.3 主试者应熟记操作方法和测查通过要求

6.1.4 主试者的位置应正确，桌面应整洁，测查工具箱内的用具不应让被试儿童看到，用一件取一件，用完后放回。

6.1.5 主试者应经过专业培训获得相关资质才能施测。

6.2 结果解释

6.2.1 应由受过专业培训的主试者结合儿童的综合情况对其发育行为水平予以解释和判断。

6.2.2 主试者应恰当地向家长解释儿童发育行为水平，尤其是对于发育落后的儿童更要慎重。

附 录 A
（规范性附录）
0 岁～6 岁儿童发育行为评估量表（儿心量表-Ⅱ）

0岁～6岁儿童发育行为评估量表见表A.1、图A.1、图A.2和表A.2。

表A.1 0 岁～6 岁儿童发育行为评估量表（儿心量表-Ⅱ）

项目	1 月龄	2 月龄	3 月龄	4 月龄	5 月龄
大 运 动	□1 抬肩坐起头竖直片刻	□11 拉腕坐起头竖直短时	□21 抱直头稳	□30 扶腋可站片刻	□40 轻拉腕部即坐起
	□2 俯卧头部翘动	□12 俯卧头抬离床面	□22 俯卧抬头 45°	□31 俯卧抬头 90°	□41 独坐头身前倾
精细动作	□3 触碰手掌紧握拳	□13 花铃棒留握片刻	□23 花铃棒留握 30s	□32 摇动并注视花铃棒	□42 抓住近处玩具
	□4 手的自然状态	□14 拇指轻叩可分开*	□24 两手搭在一起	□33 试图抓物	□43 玩手
适应能力	□5 看黑白靶*	□15 即刻注意大玩具	□25 即刻注意胸前玩具	□34 目光对视*	□44 注意小丸
	□6 眼跟红球过中线	□16 眼跟红球上下移动*	□26 眼跟红球 180°	□35 高声叫[R]	□45 拿住一积木注视另一积木
语 言	□7 自发细小喉音[R]	□17 发 a、o、e 等母音[R]	□27 笑出声[R]	□36 伊语作声[R]	□46 对人及物发声[R]
	□8 听声音有反应*	□18 听声音有复杂反应		□37 找到声源	
社会行为	□9 对发声的人有注视	□19 自发微笑[R]	□28 见人会笑	□38 注视镜中人像	□47 对镜有游戏反应
	□10 眼跟踪走动的人	□20 逗引时有反应	□29 灵敏模样	□39 认亲人[R]	□48 见食物兴奋[R]
项目	6 月龄	7 月龄	8 月龄	9 月龄	10 月龄
大 运 动	□49 仰卧翻身[R]	□59 悬垂落地姿势*	□68 双手扶物可站立	□77 拉双手会走	□86 保护性支撑*
	□50 会拍桌子	□60 独坐直	□69 独坐自如	□78 会爬	□87 自己坐起
精细动作	□51 会撕揉纸张	□61 耙弄到小丸	□70 拇他指捏小丸	□79 拇食指捏小丸	□88 拇食指动作熟练
	□52 耙弄到桌上一积木	□62 自取一积木，再取另一块	□71 试图取第三块积木	□80 从杯中取出积木	
适应能力	□53 两手拿住积木	□63 积木换手	□72 有意识地摇铃	□81 积木对敲	□89 拿掉扣积木杯玩积木
	□54 寻找失落的玩具	□64 伸手够远处玩具	□73 持续用手追逐玩具	□82 拨弄铃舌	□90 寻找盒内东西

语　　言	□55 叫名字转头	□65 发 da-da、ma-ma 等无所指[R]	□74 模仿声音[R]	□83 会欢迎[R]	□91 模仿发语声[R]
	□56 理解手势		□75 可用动作手势表达（2/3）[R]	□84 会再见[R]	
社会行为	□57 自喂食物[R]	□66 抱脚玩	□76 懂得成人面部表情	□85 表示不要[R]	□92 懂得常见物及人名称
	□58 会躲猫猫	□67 能认生人[R]			□93 按指令取东西
项目	11 月龄	12 月龄	15 月龄	18 月龄	21 月龄
大 运 动	□94 独站片刻	□103 独站稳	□112 独走自如	□120 扔球无方向	□128 脚尖走[R]
	□95 扶物下蹲取物	□104 牵一手可走			□129 扶楼梯上楼
精细动作	□96 积木放入杯中	□105 全掌握笔留笔道	□113 自发乱画	□121 模仿画道道	□130 水晶线穿扣眼
		□106 试把小丸投小瓶	□114 从瓶中拿到小丸		□131 模仿拉拉锁
适应能力	□97 打开包积木的方巾	□107 盖瓶盖	□115 翻书两次	□122 积木搭高四块	□132 积木搭高 7~8 块
	□98 模仿拍娃娃		□116 盖上圆盒	□123 正放圆积木入型板	□133 知道红色
语　　言	□99 有意识地发一个字音[R]	□108 叫爸爸妈妈有所指[R]	□117 会指眼耳鼻口手	□124 懂得三个投向	□134 回答简单问题
	□100 懂得“不”[R]	□109 向他/她要东西知道给	□118 说 3～5 个字[R]	□125 说十个字词[R]	□135 说 3～5 个字的句子[R]
社会行为	□101 会从杯中喝水[R]	□110 穿衣知配合[R]	□119 会脱袜子[R]	□126 白天能控制大小便[R]	□136 能表示个人需要[R]
	□102 会摘帽子	□111 共同注意[R]		□127 会用匙[R]	□137 想象性游戏[R]
项目	24 月龄	27 月龄	30 月龄	33 月龄	36 月龄
大 运 动	□138 双足跳离地面	□146 独自上楼	□156 独脚站 2s	□165 立定跳远	□174 双脚交替跳
		□147 独自下楼			
精细动作	□139 穿过扣眼后拉线	□148 模仿画竖道	□157 穿扣子 3～5 个	□166 模仿画圆	□175 模仿画交叉线
		□149 对拉锁	□158 模仿搭桥	□167 拉拉锁	□176 会拧螺丝
适应能力	□140 一页页翻书	□150 认识大小	□159 知道 1 与许多	□168 积木搭高 10 块	□177 懂得“3”
	□141 倒放圆积木入型板	□151 正放型板	□160 倒放型板	□169 连续执行三个命令	□178 认识两种颜色
语　　言	□142 说两句以上诗或儿歌	□152 说 7~10 个字的句子	□161 说出图片 10 样	□170 说出性别	□179 说出图片 14 样
	□143 说常见物用途(碗笔凳球)	□153 理解指令	□162 说自己名字	□171 分清“里”“外”	□180 发音基本清楚
社会行为	□144 会打招呼	□154 脱单衣或裤[R]	□163 来回倒水不洒	□172 会穿鞋	□181 懂得“饿了、冷了、累了”
	□145 问“这是什么？”[R]	□155 开始有是非观念	□164 女孩扔果皮	□173 解扣子	□182 扣扣子

项目	42 月龄	48 月龄	54 月龄	60 月龄	66 月龄
大 运 动	□183 交替上楼	□193 独脚站 5s	□203 独脚站 10s	□213 单脚跳	□222 接球
	□184 并足从楼梯末级跳下	□194 并足从楼梯末级跳下稳	□204 足尖对足跟向前走 2m	□214 踩踏板	□223 足尖对足跟向后走 2m
精细动作	□185 拼圆形、正方形	□195 模仿画方形	□205 折纸边角整齐	□215 照图拼椭圆形	□224 会写自己的名字
	□186 会用剪刀	□196 照图组装螺丝	□206 筷子夹花生米	□216 试剪圆形	□225 剪平滑圆形
适应能力	□187 懂得“5”	□197 找不同（3 个）	□207 类同	□217 找不同（5 个）	□226 树间站人
	□188 认识四种颜色	□198 图画补缺（3/6）	□208 图画补缺（4/6）	□218 图画补缺（5/6）	□227 十字切苹果
语　　言	□189 会说反义词	□199 模仿说复合句	□209 会漱口	□219 你姓什么？	□228 知道自己属相
	□190 说出图形（△○□）	□200 锅、手机、眼睛的用途	□210 会认识数字	□220 说出两种圆形的东西	□229 倒数数字
社会行为	□191 会穿上衣[R]	□201 会做集体游戏[R]	□211 懂得上午、下午	□221 你家住哪里？	□230 为什么要走人行横道？
	□192 吃饭之前为什么要洗手？	□202 分辨男女厕所	□212 数手指		□231 鸡在水中游

项目	72 月龄	78 月龄	84 月龄
大 运 动	□232 抱肘连续跳	□242 踢带绳的球	□252 连续踢带绳的球
	□233 拍球（2 个）	□243 拍球（5 个）	□253 交替踩踏板
精细动作	□234 拼长方形	□244 临摹六边形	□254 学翻绳
	□235 临摹组合图形	□245 试打活结	□255 打活结
适应能力	□236 找不同（7 个）	□246 图形类比	□256 数字类比
	□237 知道左右	□247 面粉的用途	□257 什么动物没有脚？
语　　言	□238 描述图画内容	□248 归纳图画主题	□258 为什么要进行预防接种？
	□239 上班、窗、苹果、香蕉(2/3)	□249 认识钟表	□259 毛衣、裤、鞋共同点
社会行为	□240 一年有哪四个季节？	□250 懂得星期几	□260 紧急电话
	□241 认识标识	□251 雨中看书	□261 猫头鹰抓老鼠

注 1：标注[R]的测查项目表示该项目的表现可以通过询问家长获得。

注 2：标注*的测查项目表示该项目如果未通过需要引起注意。

注 3：测查床规格：长 140cm，宽 77cm，高 143cm，栏高 63cm。

注 4：测查用桌子规格：长 120cm，宽 60cm，高 75cm，桌面颜色深绿。

注 5：测查用楼梯规格：上平台：由两梯相对合成的平台，长 50cm×宽 60cm×高 50cm（距地面高度）。底座全梯：长 150cm（单梯底座长 75cm）。每一个阶梯面：长 60cm×宽 25cm×高 17cm，共 3 阶梯。单侧扶栏：长 90cm，直径 2.5cm，从梯面计算扶栏高 40cm，直径 2.5cm。

4　1　2　9　3　6　10　5　8　7

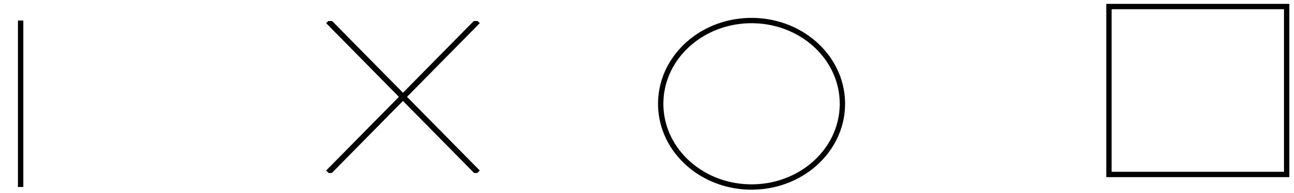

图A.1　0岁～6岁儿童发育行为评估量表（儿心量表-Ⅱ）数字识别和模仿画图测查图

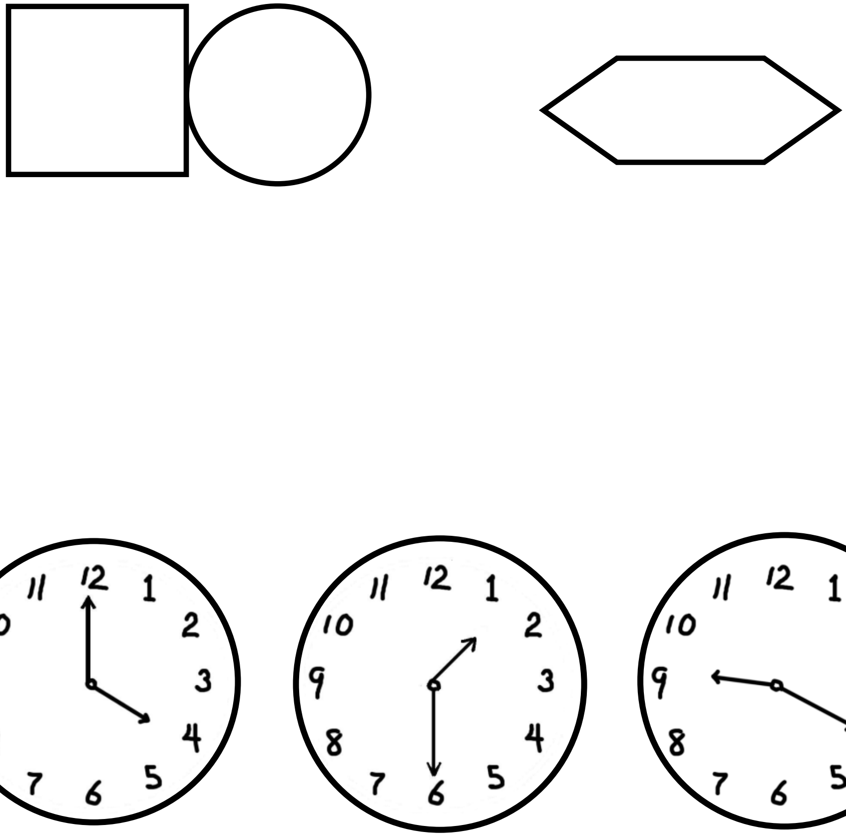

图 A.2　0 岁～6 岁儿童发育行为评估量表（儿心量表-Ⅱ）临摹图形和认识钟表测查图

表A.2　0岁～6岁儿童发育行为评估量表（儿心量表-Ⅱ）基本信息和结果记录

<table>
<tr><td>姓　　名</td><td></td><td>性　　别</td><td></td><td>民　　族</td><td></td></tr>
<tr><td>测验日期</td><td colspan="5">年　　月　　日</td></tr>
<tr><td>出生日期</td><td colspan="5">年　　月　　日</td></tr>
<tr><td>实足年龄</td><td colspan="5"></td></tr>
<tr><td>项　　目</td><td colspan="2">智　龄（月）</td><td colspan="3">发育商（DQ）</td></tr>
<tr><td>大 运 动</td><td colspan="2"></td><td colspan="3"></td></tr>
<tr><td>精细动作</td><td colspan="2"></td><td colspan="3"></td></tr>
<tr><td>适应能力</td><td colspan="2"></td><td colspan="3"></td></tr>
<tr><td>语　　言</td><td colspan="2"></td><td colspan="3"></td></tr>
<tr><td>社会行为</td><td colspan="2"></td><td colspan="3"></td></tr>
<tr><td>全 量 表</td><td colspan="2"></td><td colspan="3"></td></tr>
</table>

主试者：

附 录 B
（规范性附录）
0 岁～6 岁儿童发育行为评估量表（儿心量表-Ⅱ）操作方法和测查通过要求

0岁～6岁儿童发育行为评估量表操作方法和测查通过要求见表B.1。

表B.1 0 岁～6 岁儿童发育行为评估量表（儿心量表-Ⅱ）操作方法和测查通过要求

测查项目	操作方法	测查通过要求
1. 抬肩坐起头竖直片刻	婴儿仰卧，主试者面向婴儿站立，对婴儿微笑、说话，直到婴儿注视到主试者的脸。这时主试者轻轻握住婴儿双肩（四指并拢置于肩胛骨外侧，食指不能触碰颈部），将婴儿拉坐起来，观察婴儿控制头的能力	婴儿头可竖直保持2s或以上
2. 俯卧头部翘动	婴儿俯卧，前臂屈曲支撑，用玩具逗引婴儿抬头，观察其反应	婴儿有头部翘动即可通过
3. 触碰手掌紧握拳	婴儿仰卧，主试者将食指从尺侧放入婴儿手掌中	婴儿能将拳头握紧
4. 手的自然状态	主试者观察婴儿清醒时手的自然状态	双手拇指内收不达掌心，无发紧即通过
5. 看黑白靶*	婴儿仰卧，主试者将黑白靶拿在距婴儿脸部上方20cm处移动，吸引婴儿注意	婴儿眼睛可明确注视黑白靶
6. 眼跟红球过中线	婴儿仰卧，主试者手提红球，在婴儿脸部上方20cm处轻轻晃动以引起婴儿注意，然后把红球慢慢移动，从头的一侧沿着弧形，移向中央，再移向头的另一侧，观察婴儿头部和眼睛的活动。	当主试者把红球移向中央时，婴儿用眼睛跟踪看着红球转过中线，三试一成
7. 自发细小喉音^{R}	婴儿仰卧、清醒。注意其发音	观察或询问，小儿能发出任何一种细小柔和的喉音
8. 听声音有反应*	婴儿仰卧，在其一侧耳上方10cm～15cm处轻摇铜铃，观察婴儿的反应。（双侧均做，一侧通过即可）	婴儿听到铃声有一种或多种反应
9. 对发声的人有注视	主试者面对婴儿的脸微笑并对其说话。但不能触碰婴儿的面孔或身体	婴儿能注视主试者的脸
10. 眼跟踪走动的人	婴儿横放在床上或斜躺在家长臂弯里，主试者站立（直立位，勿弯腰）逗引婴儿引起其注意后左右走动，观察婴儿眼睛是否追随主试者	眼睛随走动的人转动
11. 拉腕坐起头竖直短时	婴儿仰卧，主试者将拇指置于婴儿掌心，余四指握住腕部轻拉婴儿坐起，观察婴儿控制头部的能力	当把婴儿拉起成坐位时婴儿头可自行竖直，保持5s或以上
12. 俯卧头抬离床面	婴儿俯卧，前臂屈曲支撑，用玩具逗引婴儿抬头，观察其反应	婴儿可自行将头抬离床面达2s或以上。
13. 花铃棒留握片刻	婴儿仰卧，将花铃棒放在婴儿手中	握住花铃棒不松手达2s或以上
14. 拇指轻叩可分开*	主试者分别轻叩婴儿双手手背，观察拇指自然放松的状态	婴儿双手握拳稍紧，拇指稍内收，但经轻叩即可打开
15. 即刻注意大玩具	婴儿仰卧，用娃娃在婴儿脸部上方20cm处晃动，观察其反应。	可立刻注意到娃娃，三试一成

16. 眼跟红球上下移动*	婴儿仰卧，主试者提起红球，在婴儿脸部上方20cm处轻轻晃动以引起婴儿注意，先慢慢向上移动，然后再从头顶向下颏处移动	婴儿眼睛能上或下跟随红球
17. 发a、o、e等母音R	询问或逗引婴儿发音	能从喉部发出a、o、e等元音来
18. 听声音有复杂反应	婴儿仰卧，在其一侧耳上方10cm～15cm处轻摇铜铃，观察婴儿的反应。（双侧均做，一侧通过即可）	婴儿听到声音有表情和肢体动作的变化
19. 自发微笑R	观察或询问婴儿在无外界逗引时是否有自发微笑的情况	婴儿能自发出现微笑，但不一定出声。睡眠时微笑不通过
20. 逗引时有反应	婴儿仰卧，主试者弯腰，对婴儿点头微笑或说话进行逗引，观察其反应。但不能触碰婴儿的面孔或身体	经逗引，婴儿会出现微笑、发声、手脚乱动等一种或多种表现
21. 抱直头稳	竖抱婴儿，观察婴儿控制头部的能力	能将头举正并稳定10s或以上
22. 俯卧抬头45°	婴儿俯卧，前臂屈曲支撑，头正中位，用玩具逗引婴儿抬头，观察其反应	头可自行抬离床面，面部与床面成45°，持续5s或以上
23. 花铃棒留握30s	婴儿仰卧或侧卧，将花铃棒放入婴儿手中	婴儿能握住花铃棒30s，不借助床面的支持
24. 两手搭在一起	婴儿仰卧，主试者观察婴儿双手是否能够自发搭在一起，或主试者将其两手搭在一起，随即松手，观察婴儿双手状态。	婴儿能将双手搭在一起，保持3s～4s
25. 即刻注意胸前玩具	婴儿仰卧，主试者将娃娃在婴儿身体上方20cm处沿中线自下向上移动。当玩具到婴儿乳头连线至下颏之间时，观察婴儿反应	当娃娃移动至婴儿乳头连线至下颌之间时，立即注意即可通过
26. 眼跟红球180°	婴儿仰卧，主试者手提红球，在婴儿脸部上方20cm处轻轻晃动以引起婴儿注意，然后把红球慢慢移动，从头的一侧沿着弧形，移向中央，再移向头的另一侧，观察婴儿头部和眼睛的活动	婴儿用眼及头跟随红球转动180°，三试一成
27. 笑出声R	逗引婴儿笑，但不得接触身体	观察或询问，婴儿能发出“咯咯”笑声
28. 见人会笑	主试者面对婴儿，不做出接近性的社交行为或动作，观察婴儿在无人逗引时的表情	婴儿见到人自行笑起来
29. 灵敏模样	主试者观察婴儿在不经逗引的情况下，对周围人和环境的反应	婴儿不经逗引可观察周围环境，眼会东张西望
30. 扶腋可站片刻	主试者扶婴儿腋下，置于立位后放松手的支持，观察其反应	婴儿可用自己双腿支持大部分体重达2s或以上
31. 俯卧抬头90°	婴儿俯卧，前臂屈曲支撑，头正中位，用玩具逗引婴儿抬头，观察其反应	头可自行抬离床面，面部与床面呈90°，持续5s或以上
32. 摇动并注视花铃棒	抱坐，将花铃棒放入婴儿手中，鼓励婴儿摇动	婴儿能注视花铃棒，并摇动数下
33. 试图抓物	婴儿仰卧，将花铃棒拿到婴儿可及的范围内，观察婴儿反应，但不能触碰婴儿	婴儿手臂试图抬起或有手抓动作即可通过
34. 目光对视*	主试者或母亲对婴儿说话，观察婴儿是否与人对视	婴儿能与成人对视，并保持5s或以上

35. 高声叫[R]	观察或询问婴儿在高兴或不满时的发音	会高声叫（非高调尖叫）
36. 伊语作声[R]	观察婴儿安静时的发音	观察或询问，婴儿会类似自言自语，无音节、无意义
37. 找到声源	抱坐，主试者在婴儿耳后上方15cm处轻摇铜铃，观察其反应	可回头找到声源，一侧耳通过即可
38. 注视镜中人像	将无边镜子横放在婴儿面前约20cm处，主试者或母亲可在镜中逗引婴儿，观察婴儿反应	婴儿可经逗引或自发注视镜中人像
39. 认亲人[R]	观察婴儿在看到母亲或其他亲人或听到亲人声音后的表情变化	观察或询问，在见到母亲或其他亲人时，婴儿会变得高兴起来
40. 轻拉腕部即坐起	婴儿仰卧，主试者握住腕部，轻拉到坐的位置	婴儿自己能主动用力坐起，拉坐过程中无头部后滞现象
41. 独坐头身前倾	将婴儿以坐姿置于床上	独坐保持5s或以上，头身向前倾
42. 抓住近处玩具	抱坐，婴儿手置于桌上。玩具（如花铃棒）放在距离婴儿手掌一侧2.5cm处，鼓励婴儿取玩具	婴儿可用一手或双手抓住玩具
43. 玩手	观察婴儿能否把双手放在一起互相玩弄	婴儿会自发将双手抱到一起玩
44. 注意小丸	桌面上放一小丸，主试者指点小丸或把小丸动来动去，以引起婴儿注意	婴儿明确地注意到小丸
45. 拿住一积木注视另一积木	抱坐，婴儿手置于桌上，主试者先放一块积木在婴儿手中，再放另一块积木于桌上婴儿可及范围内，适当逗引，观察婴儿对第二块积木的反应	婴儿拿着放在手中的第一块积木，当第二块积木靠近时，目光明确地注视第二块积木
46. 对人及物发声[R]	观察或询问婴儿看到熟悉的人或玩具时的发音	观察或询问，婴儿会发出象说话般的声音，如伊伊呀呀、ma、pa、ba等辅元结合音
47. 对镜有游戏反应	将无边镜子竖放在婴儿面前约20cm处，主试者及家长影像不能在镜内出现，观察婴儿反应	对镜中自己的影像有面部表情变化或伴有肢体动作。
48. 见食物兴奋[R]	观察婴儿看到奶瓶、饼干、水等食物时的反应	观察或询问，当婴儿看到奶瓶或母亲乳房时，表现出高兴要吃的样子
49. 仰卧翻身[R]	婴儿仰卧，用玩具逗引其翻身	观察或询问，婴儿可从仰卧自行翻到俯卧位
50. 会拍桌子	抱坐，主试者示范拍打桌面，鼓励婴儿照样做	婴儿经示范后或自发拍打桌面，并拍响
51. 会撕揉纸张	将一张28g粉色打字纸放入婴儿手中，使婴儿能抓住纸，观察婴儿反应	能用双手反复揉搓纸张两次或以上，或将纸撕破
52. 耙弄到桌上一积木	抱坐，放一积木在婴儿容易够到的桌面上，观察婴儿反应	婴儿伸出手触碰到积木并抓握到

53．两手拿住积木	抱坐，先后递给婴儿两块积木，婴儿自己拿或被动放在手中均可	婴儿一手拿一块积木，保持在手里10s或以上
54．寻找失落的玩具	以红球逗引婴儿注意，红球位置应与婴儿双眼在同一水平线上。主试者手提红球，当婴儿注意到红球后，立即松手使红球落地，此时主试者的手保持原姿势，观察婴儿反应	红球落地后，婴儿立即低下头寻找红球
55．叫名字转头	主试者或家长在婴儿背后呼唤其名字，观察其反应	婴儿会转头寻找呼唤的人
56．理解手势	主试者或妈妈（带养人）伸手表示要抱，不得出声提示，观察婴儿反应	婴儿理解并将手伸向主试者或妈妈（带养人），二试一成
57．自喂食物[R]	观察或询问婴儿拿到一块饼干或其他能拿住的食物时，能否送至口中并咀嚼	能将饼干送入口中并咀嚼，有张嘴咬的动作而不是吸吮
58．会躲猫猫	主试者把自己的脸藏在一张中心有孔的A4纸后面（孔直径0.5cm），呼唤婴儿名字，婴儿听到声音，观望时，主试者沿纸边在纸的同一侧反复出现两次并逗引说“喵、喵”，第三次呼唤婴儿名字后从纸孔观察婴儿表情	第三次呼唤婴儿时，婴儿视线再次转向主试者刚才露脸的方向
59．悬垂落地姿势*	扶腋下使婴儿呈悬空位，足离床面20cm～30cm，立位瞬时落下，观察脚落地瞬时的姿势	婴儿能全脚掌着地
60．独坐直	将婴儿以坐姿置于床上	独坐时背直，无需手支撑床面，保持1min或以上
61．耙弄到小丸	抱坐，将一小丸放在桌上，鼓励婴儿取	婴儿用所有手指弯曲做耙弄、搔抓动作，最后成功地用全掌抓到小丸
62．自取一积木，再取另一块	抱坐，出示一积木给婴儿，抓住后，再出示另一块，观察其反应	婴儿主动伸手去抓桌上的积木，第一块积木握住并保留在手中后，又成功地用另一只手抓住第二块积木
63．积木换手	抱坐，出示一积木给婴儿，婴儿拿住后，再向拿积木的手前出示另一块积木，观察其反应	婴儿将第一块积木传到另一只手后，再去拿第二块积木
64．伸手够远处玩具	抱坐，将一玩具放于婴儿手恰好够不到的桌面上，观察其反应	欠身取，并能拿到玩具
65．发da-da ma-ma 无所指[R]	观察婴儿在清醒状态时的发声情况	观察或询问，婴儿会发da-da 、ma-ma的双唇音，但无所指
66．抱脚玩	婴儿仰卧，观察其是否会自发或在主试者协助下将脚放入手中后玩脚	婴儿能抱住脚玩或吸吮
67．能认生人[R]	观察或询问婴儿对陌生人的反应	婴儿有拒抱、哭、不高兴或惊奇等表现
68．双手扶物可站立	将婴儿置于床上，协助婴儿双手抓握栏杆，胸部不靠栏杆，呈站立姿势观察	双手扶栏杆支撑全身重量，保持站立位5s或以上
69．独坐自如	婴儿坐位，用玩具逗引，婴儿上身可自由转动取物，或轻轻将婴儿肩头向对侧推，观察其侧平衡	独坐时无须手支撑，上身可自由转动取物或侧推后回正保持平衡不倒

70. 拇他指捏小丸	抱坐，将一小丸放在桌上，鼓励婴儿取	婴儿会用拇他指捏起小丸
71. 试图取第三块积木	连续出示两块积木后婴儿均能拿到，再出示第三块积木鼓励婴儿取	有要取第三块积木的表现，不一定能取到，前两块仍保留在手中
72. 有意识地摇铃	主试者示范摇铃，鼓励婴儿照样做	婴儿能够有意识地摇铃
73. 持续用手追逐玩具	以玩具逗引婴儿来取，将要取到时，主试者将玩具移动到稍远的地方，观察其反应	婴儿持续追逐玩具，力图拿到，但不一定取到
74. 模仿声音[R]	观察或询问婴儿是否会模仿咳嗽、弄舌的声音	观察或询问，婴儿能模仿发出类似声音
75. 可用动作手势表达(2/3)[R]	主试者询问家长，婴儿是否常有主动伸手表示要抱；摊开手表示没有；咂咂嘴表示好吃等动作手势	三问中，有两项表现即可通过
76. 懂得成人面部表情	主试者或家长对婴儿训斥或赞许，观察其反应	婴儿表现出委屈或兴奋等反应
77. 拉双手会走	站立位，主试者牵婴儿双手，牵手时不过多给力，鼓励婴儿向前行走	婴儿可自己用力，较协调地移动双腿，向前行走三步或以上
78. 会爬	婴儿俯卧，用玩具逗引婴儿爬	婴儿能将腹部抬离床面，四点支撑向前爬行（膝手爬）
79. 拇食指捏小丸	抱坐，将一小丸放在桌上，鼓励婴儿取	婴儿会用拇食指捏起小丸
80. 从杯中取出积木	主试者在婴儿注视下将积木放入杯中，鼓励婴儿取出	婴儿能自行将积木取出，不能倒出
81. 积木对敲	主试者出示两块积木，示范积木对敲后，让婴儿一手拿一块，鼓励其照样做	婴儿能把双手合到中线，互敲积木，对击可不十分准确
82. 拨弄铃舌	主试者轻摇铜铃以引起婴儿注意，然后将铜铃递给婴儿，观察其对铜铃的反应	婴儿有意识寻找并拨弄或拿捏铃舌
83. 会欢迎[R]	主试者只说欢迎，不做手势示范，鼓励婴儿以手势表示	观察或询问，婴儿能够做出欢迎的手势
84. 会再见[R]	主试者只说再见，不做手势示范，鼓励婴儿以手势表示	观察或询问，婴儿能够做出再见的手势
85. 表示不要[R]	观察或询问婴儿对不感兴趣的物品的反应	观察或询问，婴儿对不要之物有摇头或推开的动作
86. 保护性支撑*	主试者站立在床或桌边，由婴儿背后扶持其腋下抱起，然后快速做俯冲动作，观察婴儿反应	婴儿出现双手张开，向前伸臂，类似保护自己的动作
87. 自己坐起	将婴儿置于俯卧位，用玩具逗引，观察婴儿能否坐起	无需协助，婴儿能较协调地从俯卧位坐起，并坐稳
88. 拇食指动作熟练	抱坐，将一小丸放在桌上，鼓励婴儿取	婴儿会用拇食指的指端协调、熟练且迅速地对捏起小丸
89. 拿掉扣积木杯玩积木	积木放在桌上，在婴儿注视下用杯子盖住积木，杯子的把手对着婴儿，鼓励婴儿取积木	婴儿能主动拿掉杯子，取出藏在杯子里面的积木
90. 寻找盒内东西	在婴儿面前摇响装有硬币的盒，然后避开婴儿将硬币取出，给婴儿空盒，观察其反应	婴儿能明确地寻找盒内的硬币
91. 模仿发语声[R]	观察或询问婴儿是否会模仿“妈妈”、“爸爸”、“拿”、“走”等语音	观察或询问，婴儿能模仿发语声

92．懂得常见物及人名称	主试者问婴儿 “妈妈在哪里？”“灯在哪里？”“阿姨在哪里？”等人或物的名称，观察其反应	婴儿会用眼睛注视或指出2种或以上的人或物
93．按指令取东西	将娃娃、球和杯子并排放在婴儿双手可及的桌面上，鼓励婴儿按指令取其中的一件。（每样东西交替问两次，不能连续问）	婴儿能理解指令并成功拿对其中一种或一种以上物品
94．独站片刻	将婴儿置于立位，待婴儿站稳后松开双手，观察其站立情况	婴儿能独自站立2s或以上
95．扶物下蹲取物	婴儿手扶围栏站立，不得倚靠。将玩具放在其脚边，鼓励婴儿下蹲取物	一手扶栏杆蹲下，用另一只手捡玩具，并能再站起来
96．积木放入杯中	主试者示范将积木放入杯中，鼓励婴儿照样做	婴儿能有意识地将积木放入杯中并撒开手
97．打开包积木的方巾	在婴儿注视下用方巾包起一积木，然后打开，再包上，鼓励婴儿找	婴儿有意识地打开包积木的方巾，寻找积木，成功将积木拿到手
98．模仿拍娃娃	主试者示范拍娃娃，鼓励婴儿照样做	婴儿学大人样子轻拍娃娃
99．有意识地发一个字音[R]	观察或询问婴儿有意识的发音情况	观察或询问，有意识并正确地发出相应的字音，如爸、妈、拿、走、姨、奶、汪汪等
100．懂得“不”[R]	婴儿取一玩具玩时，主试者说“不动”、“不拿”，不要做手势，观察或询问其反应	观察或询问，婴儿会停止拿取玩具的动作
101．会从杯中喝水[R]	观察或询问婴儿能否从成人拿的杯子里喝到水	观察或询问，婴儿能从杯中喝到水
102．会摘帽子	主试者将帽子戴在婴儿头上，观察其能否摘下帽子	婴儿能用单手或双手摘下帽子
103．独站稳	将小儿置于立位，待小儿站稳后松开双手，观察其站立情况	独自站立10s或以上，允许身体轻微晃动
104．牵一手可走	主试者牵小儿一只手行走，不要用力，观察其行走情况	小儿自己迈步，牵一手能协调地移动双腿，至少向前迈三步以上
105．全掌握笔留笔道	主试者示范用笔在纸上画道，鼓励小儿模仿	小儿握笔在纸上留下笔道即可
106．试把小丸投小瓶	出示一小丸及30ml广口试剂瓶，主试者拿瓶，示范并指点将小丸放入瓶内，鼓励小儿照样做	小儿捏住小丸试往瓶内投放，但不一定成功
107．盖瓶盖	瓶盖翻放在桌上，主试者示范将瓶盖盖在瓶上，鼓励小儿照样做	小儿会将瓶盖翻正后盖在瓶上
108．叫爸爸妈妈有所指[R]	观察或询问小儿见到妈妈、爸爸时，是否会有意识并准确地叫出	小儿会主动地称呼爸爸或妈妈
109．向他/她要东西知道给	将一玩具放入小儿手中，然后主试者或家长对小儿说“把某某东西给我”，不要伸手去拿，观察小儿反应	经要求，小儿把玩具主动递给主试者或家长，并主动松手
110．穿衣知配合[R]	观察或询问成人给小儿穿衣时的配合情况	穿衣时小儿合作，会有伸手、伸腿等配合动作，不一定穿进去

111．共同注意R	观察或询问，对家长指示的某一场景或过程，小儿能否与家长一起关注	小儿有共同注意过程
112．独走自如	观察小儿走路的情况	小儿行走自如，不左右摇摆，会控制步速，不惯性前冲
113．自发乱画	主试者出示纸和笔，鼓励小儿画画	小儿能用笔在纸上自行乱画
114．从瓶中拿到小丸	出示装有小丸的30ml广口试剂瓶，递给小儿，说“阿姨想要豆豆（小丸）怎么办？”或“把豆豆给妈妈”。鼓励小儿将小丸取出，但不能说倒出	小儿能将小丸拿出或倒出
115．翻书两次	主试者示范翻书，鼓励小儿照样做	做出翻书动作两次或以上
116．盖上圆盒	主试者示范将圆盒盖好，鼓励小儿照样做	小儿会将圆盒盖上，并盖严
117．会指眼耳口鼻手	主试者问小儿“眼在哪儿？”“耳在哪儿？”“鼻子在哪儿？”等，观察其反应	能正确指出3个或3个以上身体部位
118．说3～5个字R	观察或询问小儿有意识讲话的情况	有意识地说3～5个字（妈、爸除外）
119．会脱袜子R	观察或询问小儿脱袜子的方法	观察或询问，小儿能正确且有意识地脱下袜子
120．扔球无方向	主试者示范过肩扔球，鼓励小儿照样做	小儿举手过肩扔球，可无方向
121．模仿画道道	主试者示范用蜡笔画出一无方向道道，鼓励小儿模仿	小儿能画出道道，起止自如，方向不限
122．积木搭高四块	示范搭高两块积木，推倒后一块一块出示积木，鼓励小儿搭高	小儿搭高四块积木或以上，三试一成
123．正放圆积木入型板	在型板圆孔下方放一圆积木，圆孔靠近小儿身体。主试者对小儿说“这是小朋友的家（指型板面而不是圆孔），请帮这个小朋友（指圆积木）找到自己的家”，不示范	不经指点，能正确将圆积木一次性放入孔内
124．懂得三个投向	请小儿把三块积木分别递给妈妈、阿姨、放在桌子上，妈妈阿姨不能伸手要	小儿会正确地将积木送到要求的地方
125．说十个字词R	观察或询问小儿有意识讲话的情况并记录	有意识说10个或以上单字或词（爸、妈除外）
126．白天能控制大小便R	观察或询问小儿大小便控制情况，或询问白天是否尿湿裤子	经人提醒或主动示意大小便，白天基本不尿湿裤子
127．会用匙R	观察或询问小儿是否会自己用匙	小儿能自己用匙吃饭，允许少量遗洒
128．脚尖走R	主试者示范用脚尖行走，鼓励小儿照样做	小儿能用脚尖连续行走三步以上，脚跟不得着地
129．扶楼梯上楼	在楼梯上放一玩具，鼓励小儿上楼去取	小儿能扶楼梯扶手，熟练地上三阶以上台阶。
130．水晶线穿扣眼	主试者示范用水晶线穿过扣眼，鼓励小儿照样做	小儿能将水晶线穿过扣眼0.5cm以上

131．模仿拉拉锁	示范拉拉锁，拉上、拉下各一次。主试者固定拉锁两端，鼓励小儿照样做	小儿能双手配合将锁头来回移动，超过全拉锁的一半
132．积木搭高7～8块	示范搭高两块积木，推倒后一块一块出示积木，鼓励小儿搭高	小儿搭高7～8块积木，三试一成
133．知道红色	出示红、黄、蓝、绿四色图片，问小儿“哪个是红色？”	小儿能在四色图片中正确指出红色
134．回答简单问题	主试者问“这是什么（球）？”“那是谁（带小儿者）？”“爸爸干什么去了（上班）？”	小儿均能正确回答
135．说3～5个字的句子[R]	观察或询问小儿有意识说话的情况	小儿能有意识地说出3～5个字的句子，有主谓语
136．能表示个人需要[R]	观察或询问小儿是否会明确表示自己的需要	小儿会说出三种或以上的需要，如“吃饭、喝水、玩汽车、上街”等，可伴手势
137．想象性游戏[R]	观察或询问小儿是否有想象性游戏，如假装给娃娃或动物玩具喂饭、盖被子、打针等	小儿有想象性游戏
138．双足跳离地面	主试者示范双足同时离地跳起，鼓励小儿照样做	小儿会双足同时跳离地面，同时落地，两次以上
139．穿过扣眼后拉线	主试者示范用水晶线穿过扣眼，并将线拉出，鼓励小儿照样做	小儿能将水晶线穿过扣眼，并能将线拉出
140．一页页翻书	主试者示范一页页翻书，鼓励小儿照样做	小儿会用手捻书页，每次一页，连续翻书三页或以上
141．倒放圆积木入型板	在小儿能正放圆积木入型板的基础上，将型板倒转180°。圆积木仍在原处，主试者对小儿说“这是小朋友的家（指型板），请帮这个小朋友（指圆积木）找到自己的家”，不示范	型板倒转后，小儿能正确将圆积木一次性放入圆孔内
142．说两句以上诗或儿歌	鼓励小儿说唐诗或儿歌	小儿能自发或稍经提示开头后完整说出两句或以上唐诗或儿歌
143.说常见物用途(碗笔凳球)	主试者分别提问小儿碗、笔、板凳、球的用途	小儿会说出三种或以上物品的用途
144．会打招呼	示范或不示范小儿见人打招呼	小儿会自发或模仿说“你好”、“再见”等
145．问“这是什么？”[R]	观察或询问，小儿在见到某物时，是否能自发提问“这是什么？”	小儿会自发提出问题，主动问“这是什么？”
146．独自上楼	鼓励小儿不扶扶手上楼梯，可示范	不扶扶手，稳定地上楼梯三阶或以上
147．独自下楼	鼓励小儿不扶扶手下楼梯，可示范	不扶扶手，稳定地下楼梯三阶或以上
148．模仿画竖道	主试者与小儿同向，示范画一垂直线，注意测查纸张放正，鼓励小儿模仿	小儿能画竖线，长度＞2.5cm，所画线与垂直线的夹角应＜30°
149．对拉锁	出示打开的拉锁，示范将拉锁对好，鼓励小儿照样做	小儿能将拉锁头部分或全部插进锁孔
150．认识大小	主试者向小儿出示大小圆片，请小儿把大的给妈妈或阿姨	小儿会正确把大的给妈妈或阿姨，三试二成

151．正放型板	将圆、方、三角形三块积木放在与型板相应的孔旁，主试者对小儿说“这是小朋友的家（指型板），请帮这些小朋友（指三块积木）找到自己的家”，不示范。放置三角型积木方向要与型板一致	小儿能一次性正确放入相应孔内，仅等腰三角形可提示
152．说7～10个字的句子	主试者说一句话“星期天妈妈带我去公园”，可重复一遍，鼓励小儿复述	小儿能复述出7个字及以上，不影响句意表达
153．理解指令	主试者对小儿说“请举举你的手”和“请抬抬你的脚”，可重复指令一遍，但不能有示范的动作，观察小儿反应	小儿能按指令做出举手或抬脚动作
154．脱单衣或裤R	观察或询问小儿是否会自己脱上衣或裤子	小儿不用帮忙，自己脱掉单衣或单裤
155．开始有是非观念	主试者问小儿“打人对不对？”，观察小儿的反应或回答	小儿摇头或说出不对
156．独脚站2s	主试者示范用独脚站立，鼓励小儿照样做	小儿不扶任何物体可单脚站立2s或以上
157．穿扣子3～5个	主试者示范连续穿扣3～5个，鼓励小儿照样做	小儿能较熟练穿扣并拉过线3个或以上
158．模仿搭桥	示范用下面二块，上面一块共三块积木搭成有孔的桥，并保留模型，鼓励小儿照样做。主试者不得提示桥孔	小儿能搭出有孔的桥
159．知道1与许多	一块和数块积木分放两边，请小儿指出哪边是多的，再指另一边问“这是几个？”	小儿先正确指出哪一边多，后回答“是1个”
160．倒放型板	在小儿正放三块积木入型板的基础上，将型板倒转180°，三块积木仍在原处，主试者对小儿说“这是小朋友的家（指型板），请帮这些小朋友（指三块积木）找到自己的家”，不示范	小儿能一次性正确放入翻转后型板的相应孔内，仅等腰三角形可提示
161．说出图片10样	出示图片，依次指给小儿看，鼓励其说出图片名称	小儿能正确说出10样及以上。记录1.北极熊2.树叶3.小鸡4.青蛙5.螳螂6.猕猴桃7.树8.房子9.雨伞10.壶11.铅笔12.钥匙13.打印机14.刀15.电脑16.管钳17.轮船18.毛笔和砚台19.国旗20.脚21.嘴唇22.步枪23.雪花24中国结
162．说自己名字	主试者问小儿“你叫什么名字？”	小儿能正确回答自己的大名
163．来回倒水不洒	在一个无把儿的杯中注入1/3杯水，主试者示范将水倒入另一杯中，来回各倒一次，鼓励小儿照样做	小儿会将水来回倒两次，不洒水
164．女孩扔果皮	出示图片，问小儿 “乱扔垃圾是不对的，你看这个小女孩吃完的果皮应该扔哪儿？”，鼓励小儿回答	小儿能正确回答或指出应该扔垃圾筐
165．立定跳远	主试者示范跳过16开白纸（20cm宽），鼓励小儿照样做	小儿双足同时离地跳起跃过纸，不得踩到纸
166．模仿画圆	主试者示范画一圆形，鼓励小儿模仿	小儿所画圆二头相交，为闭合圆形，不能明显成角
167．拉拉锁	出示打开的拉锁，示范将拉锁对好并拉上，鼓励小儿照样做	小儿能将拉锁头全部插进锁孔，并有拉的意识

168．积木搭高10块	示范搭高二块积木，推倒后一块一块出示积木，鼓励小儿搭高。允许试三次	小儿能搭高积木10块。三试一成
169．连续执行三个命令	嘱小儿做三件事擦桌子、摇铃、把门打开，可再重复命令一遍。小儿开始做后，不能再提醒或给予暗示	小儿会做每件事情，没有遗忘任何一项，但顺序可颠倒
170．说出性别	主试者问小儿性别，若是女孩问“你是女孩还是男孩？”；若是男孩问“你是男孩还是女孩？”	小儿能正确说出自己的性别
171．分清“里”“外”	主试者将一小丸放入30毫升广口试剂瓶内问“小丸是在瓶里？还是在瓶外？”	小儿会正确说出是在里边
172．会穿鞋	主试者将小儿鞋脱下，鞋尖对着小儿，鼓励其穿上	小儿会穿进鞋并将鞋提上，不要求分左右
173．解扣子	出示娃娃，鼓励小儿解扣子，主试者应辅助小儿固定娃娃衣服	小儿会自己解开某一个扣子
174．双脚交替跳	主试者示范以高抬腿姿势原地交替跳起，鼓励小儿照样做	小儿可双足交替跳起，双脚离地5cm
175．模仿画交叉线	主试者与小儿同向示范画交叉线，鼓励小儿模仿	小儿能画出两直线并相交成角，直线线条较连续
176．会拧螺丝	主试者出示螺丝、螺母，嘱其拧上。如小儿不会，可示范	小儿能双手配合将螺丝、螺母组装起来
177．懂得“3”	主试者出示三块积木，问小儿“这是几块？”	小儿能正确说出“三块”
178．认识两种颜色	出示红、黄、蓝、绿四色图片，先从非红色开始问，避免顺口溜出，请小儿说出各为何种颜色	能正确说出两种或以上颜色
179．说出图片14样	出示图片，依次指给小儿看，鼓励其说出图片名称	小儿能正确说出14样及以上。记录1.北极熊2.树叶3.小鸡4.青蛙5.螳螂6.猕猴桃7.树8.房子9.雨伞10.壶11.铅笔12.钥匙13.打印机14.刀15.电脑16.管钳17.轮船18.毛笔和砚台19.国旗20.脚21.嘴唇22.步枪23.雪花24中国结
180．发音基本清楚	观察小儿在说话时的发音情况	小儿会发清楚大多数语音，不影响交流
181．懂得“饿了、冷了、累了”	主试者依次问“饿了怎么办？冷了怎么办？累了怎么办？”	小儿能正确回答两问或以上吃饭、穿衣、休息等
182．扣扣子	出示娃娃，鼓励小儿扣扣子，主试者应辅助小儿固定娃娃衣服	小儿能自己扣上娃娃的某一个扣子
183．交替上楼	主试者示范不扶扶手，双足交替上楼，鼓励小儿照样做	小儿上台阶交替用脚，一步一台阶，可交替上楼三阶或以上
184．并足从楼梯末级跳下	主试者示范站在楼梯末级，双足并拢跳至地面，鼓励小儿照样做	小儿双足并拢跳至地面，双足落地后两脚间距离小于10cm

185．拼圆形、正方形	主试者让小儿用4块塑料板拼圆形，用2块等边三角形板拼正方形，共限时2min	两个图形均要拼对
186．会用剪刀	主试者示范用打印纸剪一直线，鼓励小儿照样做	小儿能够剪出直线，长度大于10cm，与主剪方向角度小于15°
187．懂得“5”	主试者出示五块积木，问小儿“这是几块？”	小儿能正确说出“五块”
188．认识四种颜色	主试者出示红、黄、蓝、绿四色图片，先从非红色开始问，避免顺口溜出，请小儿说出各为何种颜色	四种颜色全部答对
189．会说反义词	主试者分别问（1）火是热的，冰呢？（2）大象的鼻子是长的，小兔的尾巴呢？（3）头发是黑的，牙齿呢？（4）木头是硬的，棉花呢？	四题中答对两个或以上
190．说出图形（△○□）	主试者依次出示积木△○□，问小儿“这是什么形状？”	小儿能正确回答三个图形的名称
191．会穿上衣[R]	观察小儿是否会穿上衣	小儿无需大人帮忙，会穿上衣并将扣子扣好或拉锁拉好
192.吃饭之前为什么要洗手？	主试者问小儿“吃饭之前为什么要洗手”？	小儿能回答出原因“为避免生病”等
193．独脚站5s	主试者示范用独脚站立，鼓励小儿照样做	小儿独脚站立5s或以上，身体稳定
194．并足从楼梯末级跳下稳	主试者示范站在楼梯末级，双足并拢跳至地面，鼓励小儿照样做	小儿双足并拢跳至地面，双足落地后两脚间距离小于5cm，并站稳
195．模仿画方形	主试者示范画一正方形，鼓励小儿模仿	小儿能基本模仿画出，所画图形允许稍有倾斜，有一个角可以＜45°
196．照图组装螺丝	主试者出示组装好的螺丝图片5s后收起，将分开的螺丝、平垫和螺母交给小儿，请小儿凭记忆组装。主试者可针对落下的零件提示“还有呢？”	小儿无需提示或稍经提示后自行将螺丝、平垫、螺母按顺序组装起来
197．找不同（3个）	出示找不同图画，主试者问小儿两张图画有什么不同之处？小熊示教，限时2min	能找到包括示教内容的3处不同或以上
198．图画补缺（3/6）	出示补缺图片，主试者问小儿各图中缺什么？第一幅图示教	要求说对包括示教内容的三幅图或以上
199．模仿说复合句	主试者说一句话“妈妈叫我一定不要和小朋友打架”，可重复一遍，鼓励小儿复述	小儿能够复述较完整的复合句，偶尔漏字/错字
200．锅、手机、眼睛的用途	主试者问（1）锅是做什么用的？（2）手机是干什么用的？（3）眼睛有什么作用？	三问均正确。
201．会做集体游戏[R]	观察或询问小儿能否做集体游戏	小儿能主动参加集体游戏，并能遵守游戏规则
202．分辨男女厕所	出示男女厕所标识图片，问小儿应该进哪个厕所，并提问“为什么”	小儿能正确识别标志并用语言表达出性别意义

203．独脚站10s	主试者示范用独脚站立，鼓励小儿照样做	小儿独脚站立10s或以上，身体稳定
204．足尖对足跟向前走2m	主试者示范，脚跟对脚尖向前走直线，鼓励小儿照样做	小儿能脚跟对脚尖向前走2m（六步），允许身体有小幅晃动
205．折纸边角整齐	主试者示范用一长方形纸横竖对齐各折一次，鼓励小儿照样做	小儿折纸基本成长方形，折纸边差距＜1cm，纸边夹角＜15°
206．筷子夹花生米	主试者鼓励小儿用筷子夹花生米，从桌子上夹到盒子里，连做三遍	小儿熟练地夹起三次以上，过程中无掉落
207．类同	主试者给小儿一个圆形扣子，然后出示第一组模板（包括圆型、方型、三角型），问“你手里的东西和我这些东西哪些是一类的？为什么？”然后收起，再出示第二组模版（包括方型钮扣、三角型、方型），提问同上	两问均答对
208．图画补缺（4/6）	出示补缺图片，主试者问小儿各图中缺什么？第一幅图示教	要求说对包括示教内容的四幅图或以上
209．会漱口	观察小儿是否会漱口	小儿能灵活左右漱口并将水吐出
210．会认识数字	主试者出示图片，随意指出10以内数字，让小儿认	小儿全部正确答出
211．懂得上午、下午	如在上午测试，主试者问（1）现在是上午还是下午？（2）太阳落山是在下午还是上午？　如在下午测试，则主试者问（1）现在是下午还是上午？（2）太阳升起是在上午还是下午？	两问均回答正确
212．数手指	主试者问小儿一只手有几个手指，如答对，再问两只手有几个手指	小儿会心算出两手有十个手指
213．单脚跳	主试者示范原地单脚跳，鼓励小儿照样做	小儿能单脚连续跳3次或以上，可伸开双臂保持平衡，允许小儿在一脚范围内跳动
214．踩踏板	主试者示范在一级台阶上以同一只脚上下台阶，鼓励小儿照样做	小儿以同一只脚能稳当并较熟练地完成3组，可稍有停顿
215．照图拼椭圆形	将事先画好的椭圆形放在小儿面前，瞩其将6块塑料片按图分别放进去，不予提醒，限时2min	小儿全部拼对
216．试剪圆形	主试者给小儿出示一张已画好圆形（直径7.5cm米）的1/2 A4打印纸，鼓励小儿将圆形剪下（附原图）	小儿能剪出大致圆形，允许出角
217．找不同5个	出示找不同图画，主试者问小儿两张图画有什么不同之处？小熊示教。限时2min	能找到包括示教内容的5处不同或以上
218．图画补缺（5/6）	出示补缺图片，主试者问小儿各图中缺什么？第一幅图示教	要求说对包括示教内容的五幅图或以上
219．你姓什么？	主试者问小儿“你姓什么？”	小儿正确回答出姓，连名带姓不能通过
220．说出两种圆形的东西	主试者让小儿说出两种圆形的东西	小儿能说出两种或以上圆形的东西

221．你家住哪里？	主试者问小儿“你家住在哪里？”，或追问“你再说详细些，我怎么送你回家呢？”	小儿说出的住址可使他人较容易找到
222．接球	主试者示范用双手而非前胸接球，然后与小儿相距一米，将球拍给小儿，鼓励小儿用手接住球	小儿用手接住球，三次中接住一次即可，用双臂或用前胸接球不通过
223．足尖对足跟向后走2m	主试者示范，脚跟对脚尖向后走直线，鼓励小儿照样做	小儿能脚跟对脚尖向后走2m（六步），允许身体有小幅晃动
224．会写自己的名字	主试者让小儿写出自己的名字	小儿能正确写出自己的名字。
225．剪平滑圆形	主试者给小儿出示一张已画好圆形（直径7.5cm）的1/2 A4打印纸，鼓励小儿将圆形剪下（附原图）	小儿能剪出平滑的圆形，无成角、毛边
226．树间站人	主试者问小儿“两棵树之间站一个人，一排三棵树之间站几个人？”	小儿回答“两个人。”
227．十字切苹果	主试者问小儿“将一个苹果十字切开是几块？”如小儿不理解，主试者可用手势比划提示	不经提示或仅在主试者手势比划提示后答“四块”
228．知道自己属相	主试者问小儿“你是属什么的？”	小儿能正确说出自己的属相
229．倒数数字	主试者先示教“你会倒着数数吗?1、2、3倒数就是……3、2、1，现在请你从24开始倒数，24、23、22、21……”，鼓励小儿完成倒数	小儿能较流利地正确数出13～1
230.人为什么要走人行横道？	主试者问小儿:“过马路为什么要走人行横道?”	小儿能正确回答。为了安全，如怕被汽车撞了等
231．鸡在水中游	出示鸡在水中游图画，主试者问小儿画的对不对，如回答“不对”，问哪里画错了	小儿能正确回答鸡不能在水里游泳
232．抱肘连续跳	主试者示范原地抱肘单脚跳，鼓励小儿照样做	小儿抱肘单脚原地连续跳3次或以上，基本在原地跳动
233．拍球2个	主试者示范拍球，鼓励小儿照样做（向下扔落地的第一下不算拍球）。允许试三次	小儿连续拍球2个或以上
234．拼长方形	主试者让小儿用2块非等边三角形板拼长方形，出示时要求短边相对，限时2min	小儿拼对长方形
235．临摹组合图形	主试者出示正方形和圆形的组合图形，鼓励小儿临摹。	小儿能画出，无转向
236．找不同（7个）	出示找不同图画，主试者问小儿两张图画有什么不同之处？小熊示教。限时2min	能找到包括示教内容的7处不同或以上
237．知道左右	主试者让小儿用左手摸右耳朵，右手摸左耳朵，右手摸右腿	小儿全部做对
238．描述图画内容	主试者出示三幅连环画，然后对小儿说“这三幅图连起来讲了一个故事，请你给我讲一讲故事的内容是什么？小猴子为什么哭了？”若小儿回答第一问后不再答，可再追问“小猴子为什么哭了？”	能分别描述每张图画的基本内容

239.上班，窗，苹果、香蕉(2/3)	主试者问（1）人为什么要上班？—挣钱或建设国家（2）房子为什么要有窗户？—透光或通风（3）苹果和香蕉有什么共同点？—水果	答对两题或以上。（1）挣钱或建设国家；（2）透光或通风；（3）水果
240．一年有哪四个季节？	主试者问小儿一年有哪四个季节	春、夏、秋、冬，顺序可以颠倒
241．认识标识	依次出示两组标识图片，问“哪一个是代表危险的标志？为什么？”	两组图均正确指出危险的标志，并说对理由
242．踢带绳的球	主试者示范用一手提绳，将球停稳，以内踝及足弓内侧来踢球，鼓励小儿照样做。如小儿用足外侧踢，可示范更正一次姿势	小儿连续用足内踝踢球2个或以上
243．拍球（5个）	主试者示范拍球，鼓励小儿照样做（向下扔落地的第一下不算拍球）。允许试三次	小儿连续拍球5个或以上
244．临摹六边形	主试者出示六边形图形，鼓励小儿临摹	小儿可临摹出六边形，6个角均画得好，连接线平直
245．试打活结	出示一双筷子和一根绳，主试者示范用绳将筷子以活结方式捆上，鼓励小儿照样做。小儿打结时主试者应辅助固定筷子	经示范后，小儿能用活结将筷子捆上
246．图形类比	主试者出示图形，问右边的4幅图中哪一幅放在左边空白处合适。第一题示教	小儿能指对包括第一题在内的三道题或以上
247．面粉的用途	主试者问小儿“面粉能做哪些东西？”	小儿能回答两种或以上
248．归纳图画主题	主试者出示三幅连环画，然后对小儿说“这三幅图连起来讲了一个故事，请你给我讲一讲故事的内容是什么？小猴子为什么哭了？”若小儿回答第一问后不再答，可再追问“小猴子为什么哭了？”	能明确理解故事的主题
249．认识钟表	主试者请小儿看钟表图辨认时间	小儿能辨认两张图或以上所表示的时间
250．懂得星期几	主试者先告诉小儿今天是星期几，然后提问“请告诉我后天是星期几？明天是星期几？”	小儿均能正确说出
251．雨中看书	出示雨中看书图片，主试者问小儿画的对不对，如回答“不对”，问哪里画错了	小儿能正确回答下雨了，不能在雨里看书，会淋湿、生病、书湿了
252．连续踢带绳的球	主试者示范用一手提绳，将球停稳，以内踝及足弓内侧来踢球，鼓励小儿照样做。如小儿用足外侧踢，可示范更正一次姿势	小儿用足内踝踢球3个或以上，踢一下落地一下
253．交替踩踏板	主试者示范在一级台阶上交替换脚上下共3组（示范时主试者要边喊口号边示范），请小儿照样做，若小儿不会两脚交替可提醒小儿“换脚”	小儿能稳当并较熟练地两脚交替完成3组，可稍有停顿
254．学翻绳	主试者示范将一根绳子做翻绳最初级模式，鼓励小儿跟着做	小儿能跟着主试者一步一步，或在主试者示范后自行做到中指挑绳

255．打活结	出示一双筷子和一根绳，鼓励其用绳将筷子以活结方式捆上，小儿打结时主试者应辅助固定筷子	无需示范，小儿能用活结将筷子捆上
256．数字类比	主试者出示图形，问下边的4幅图中哪一幅放在上边空白处合适。第一题示教	小儿能指对包括第一题在内的三道题或以上
257．什么动物没有脚?	主试者问小儿“什么动物没有脚？”（脚定义为走路用的）	小儿回答蛇、鱼等两类或以上没有脚的动物
258．为什么要进行预防接种	主试者问小儿“小朋友为什么要打预防针？”	小儿能表达出预防生病/感冒或打预防针可以不生病等
259．毛衣、裤、鞋共同点?	主试者问小儿“毛衣、长裤和鞋有什么共同之处？”	小儿回答都是穿的、能保暖
260．紧急电话	主试者分别问小儿火警、匪警（找警察帮助）、急救电话是多少？	小儿能正确回答出两种或以上电话号码
261．猫头鹰抓老鼠	出示猫头鹰抓老鼠图片，主试者问小儿画的对不对，如回答“不对”，问哪里画错了	小儿能正确回答猫头鹰白天睡觉，不会在白天出来抓老鼠

注 1：标注^{R}的测查项目表示该项目的表现可以通过询问家长获得。

注 2：标注*的测查项目表示该项目如果未通过需要引起注意。
